# Clerodane Diterpenes from *Casearia corymbosa* as Allosteric GABA_A_ Receptor Modulators

**DOI:** 10.1021/acs.jnatprod.1c00840

**Published:** 2022-04-27

**Authors:** Nova Syafni, Maria Teresa Faleschini, Aleksandra Garifulina, Ombeline Danton, Mahabir P. Gupta, Steffen Hering, Matthias Hamburger

**Affiliations:** † Pharmaceutical Biology, Department of Pharmaceutical Sciences, University of Basel, Klingelbergstrasse 50, 4056 Basel, Switzerland; ∥ Faculty of Pharmacy and Sumatran Biota Laboratory, Andalas University, Kampus Limau Manis, Padang, West Sumatra 25175, Indonesia; § Division of Pharmacology and Toxicology, Department of Pharmaceutical Sciences, University of Vienna, Pharmaziezentrum, Althanstrasse 14, 1090 Vienna, Austria; ⊥ Center for Pharmacognostic Research on Panamanian Flora, Faculty of Pharmacy, University of Panama, Panama City 0801, Panama

## Abstract

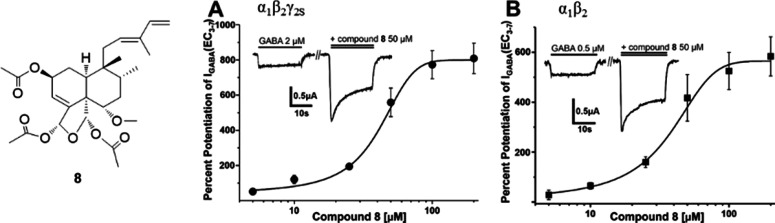

An EtOAc extract of *Casearia corymbosa* leaves led to an allosteric potentiation of the GABA signal in a fluorometric imaging plate reader (FLIPR) assay on Chinese hamster ovary (CHO) cells stably expressing GABA_A_ receptors with an α_1_β_2_γ_2_ subunit composition. The activity was tracked by HPLC-based activity profiling, and four known (**2**, **3**, **4**, and **8**) and five new clerodane-type diterpenoids (**1**, **5**–**7**, and **9**) were isolated. Compounds **1**–**8** were obtained from the active time window. The absolute configuration of all compounds was established by ECD. Compounds **3**, **7**, and **8** exhibited EC_50_ values of 0.5, 4.6, and 1.4 μM, respectively. To explore possible binding sites at the receptor, the most abundant diterpenoid **8** was tested in combination with diazepam, etazolate, and allopregnanolone. An additive potentiation of the GABA signal was observed with these compounds, while the effect of **8** was not inhibited by flumazenil, a negative allosteric modulator at the benzodiazepine binding site. Finally, the activity was validated in voltage clamp studies on *Xenopus laevis* oocytes transiently expressing GABA_A_ receptors of the α_1_β_2_γ_2_S and α_1_β_2_ subtypes. Compound **8** potentiated GABA-induced currents with both receptor subunit compositions [EC_50_ (α_1_β_2_γ_2_S) = 43.6 μM; *E*_max_ = 809% and EC_50_ (α_1_β_2_) = 57.6 μM; *E*_max_ = 534%]. The positive modulation of GABA-induced currents was not inhibited by flumazenil, thereby confirming an allosteric modulation independent of the benzodiazepine binding site.

The major inhibitory neurotransmitter in the mammalian central nervous system (CNS) is γ-aminobutyric acid (GABA).^1,2^ GABA type A (GABA_A_) receptors play an important role in modulating excitatory signals in the CNS. These are ion channels, which, upon opening, are permeable to chloride ions. A total of 19 GABA_A_ receptor subunits have been identified [six α, three β, three γ, δ, ε, θ, and π, and three ρ subunits], which assemble to numerous pentamers. These subtypes differ with respect to their distribution in the CNS and in their sensitivity to various ligands.^3^ The most abundant GABA_A_ receptor subtype consists of two α_1_ subunits, two β_2_ subunits, and one γ_2_ subunit. Besides binding sites for the endogenous neurotransmitter, GABA_A_ receptors possess several allosteric binding sites, such as the benzodiazepine, barbiturate, alcohol, and neurosteroid binding sites.^4−6^ GABA_A_ receptors are the target for anxiolytic, hypnotic, anesthetic, and anticonvulsant drugs.^6^

Various assay formats have been exploited for the discovery of GABA_A_ receptor ligands, such as radioimmunoassays, fluorescent labeling, radioactivity-based flux, and microphysiometry assays.^4,7−9^ Two-microelectrode electrophysiological assays with *Xenopus laevis* oocytes transiently expressing the GABA_A_ receptors of desired subunit composition have been used widely for the functional assessment of allosteric modulators, and larval zebrafish locomotor assays have served for the in vivo characterization of natural products.^10−13^ We recently validated a FLIPR (fluorometric imaging plate reader) assay for the screening of plant extract libraries and the discovery of allosteric GABA_A_ receptor modulators in such extracts.^14^ The assay allows for an observation of real-time membrane potential changes associated with activation of the ion channel.^15^ Chinese hamster ovary (CHO) cells stably expressing the GABA_A_ receptor with the α_1_β_2_γ_2_ subunit composition were used.^16^

*Casearia corymbosa* Kunth. (Salicaceae) is a tree distributed in the Mesoamerican region between Mexico and Venezuela. So far, clerodane-type diterpenes possessing insect antifeedant and other biological activities have been identified from this species.^17^ We here report on the activity-directed identification of diterpenoids acting as allosteric GABA_A_ receptor modulators and on experiments characterizing the allosteric binding site of the major active compound.

## Results and Discussion

**Figure 1:**
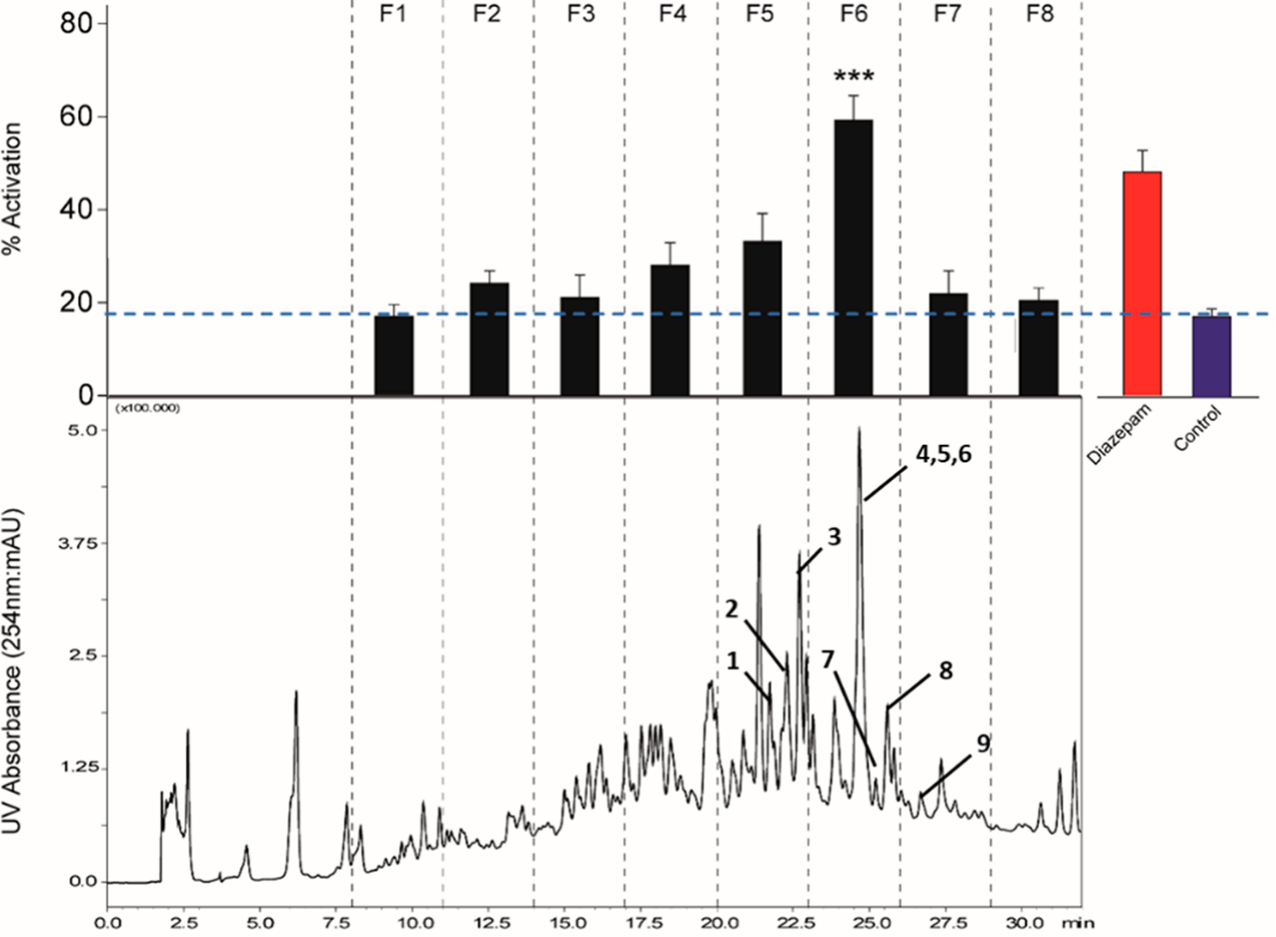
HPLC-based activity profile of the EtOAc extract. The lower panel shows the HPLC chromatogram (UV 254 nm) and the % potentiation of the GABA signal by microfractions F1–F8 is given above (*n* = 8, means ± SEM). The red and blue bars represent activation by 20 μM diazepam (in the presence of 2 μM GABA) and by 2 μM GABA (control), respectively. Dashed lines indicate microfractions collected for the bioassay (3 min each), and the numbers correspond to compounds **1** to **9**. *** Statistical significance *p* ≤ 0.001.

A library of 708 ethyl acetate extracts from plants was screened with the aid of a recently validated FLIPR assay using stably transfected CHO cells expressing GABA_A_ receptors of α_1_β_2_γ_2_ subunit composition.^14^ The EtOAc extract from leaves of *Casearia corymbosa* potentiated the GABA signal in a concentration-dependent manner, reaching >75% at 20 μg/mL (Figure S1, Supporting Information). HPLC-based activity profiling^17,18^ of the active extract enabled the localization of the main activity in microfraction F6 and, to a lesser extent, in F5 (Figure 1).

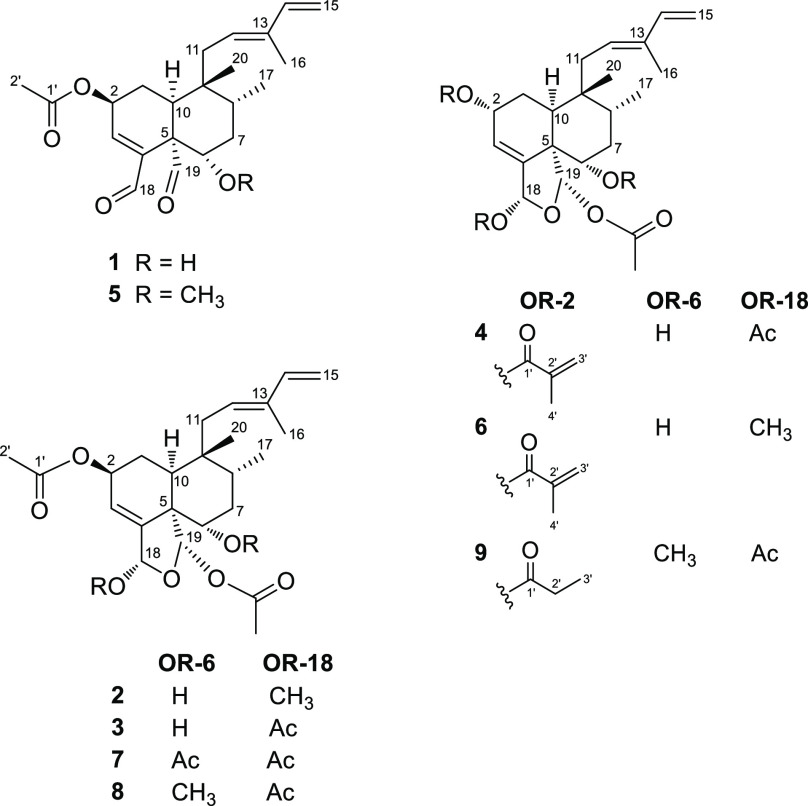



The extract was submitted to preparative column chromatography on silica gel, and 20 fractions were collected. All fractions were analyzed by HPLC-PDA-ELSD-ESIMS, whereby fractions 8–10 were found to contain compounds localized in the active time windows. These fractions were further separated by semipreparative HPLC, and compounds **1**–**9** were obtained. Of these, **1**–**8** were located in the active time windows of F5 and F6 (Figure 1).

Four compounds corresponded to the previously reported graveospene H (**2**) and corymbotins A (**8**), D (**3**), and F (**4**).^17,20^ They were all clerodane-type diterpenes with a decalin moiety, a branched side chain at C-9, and four remaining carbons attached to C-4, C-5, C-8, and C-9.^21^ However, the absolute configurations of **3**, **4**, and **8** have not been reported previously. A comparison of their ECD spectra with those of clerodane-type diterpenes from *Casearia graveolens*(20) established the absolute configurations of **3** and **8** as (2*S*,5*S*,6*S*,8*R*,9*R*,10*S*,18*R*,19*S*) and as (2*R*,5*S*,6*S*,8*R*,9*R*,10*S*,18*R*,19*S*) for compound **4** (Figures S27 and S28, Supporting Information).

**Figure 2:**
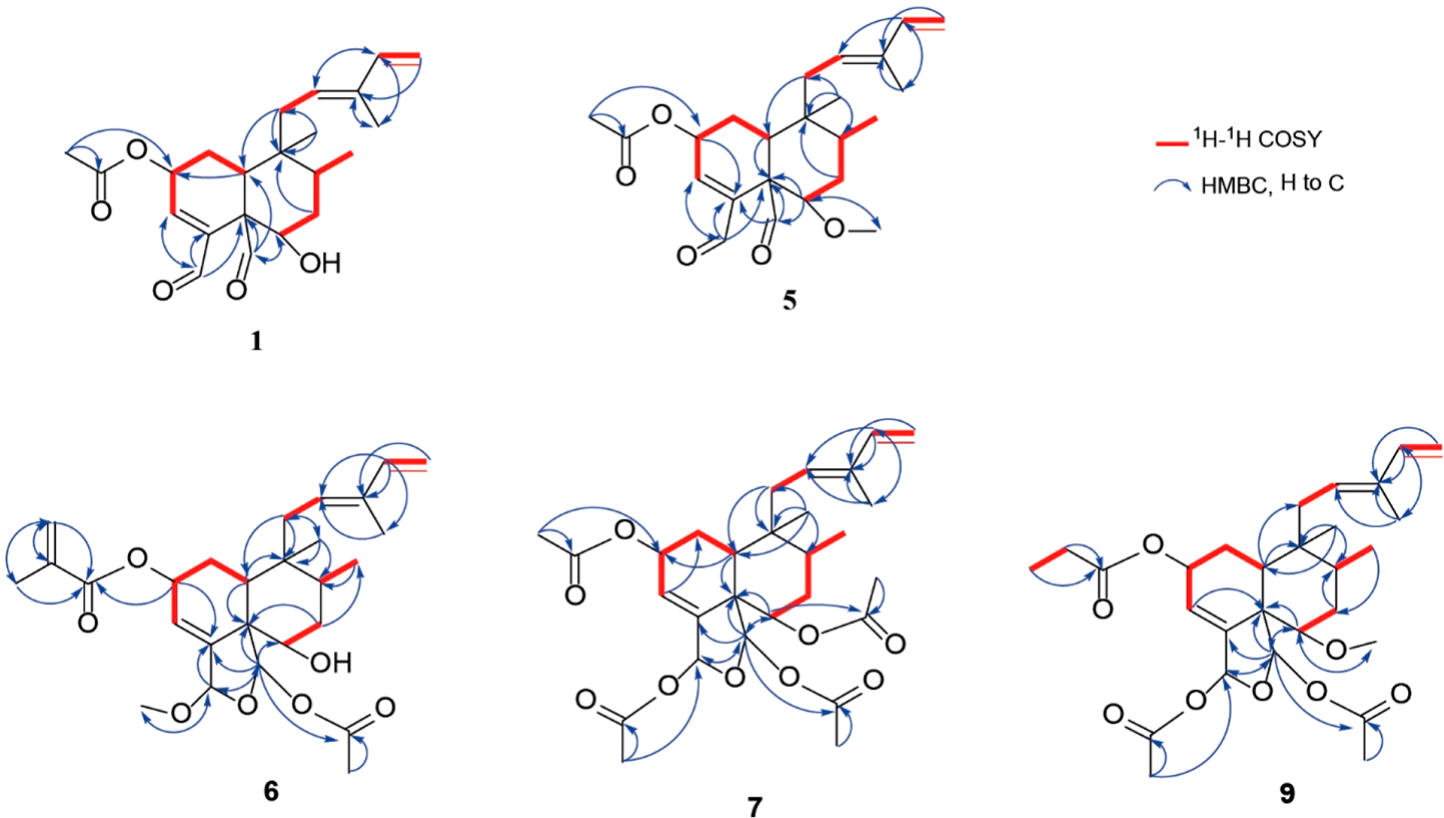
Key correlations from COSY and HMBC of compounds **1**, **5**–**7**, and **9**.

**Figure 3:**
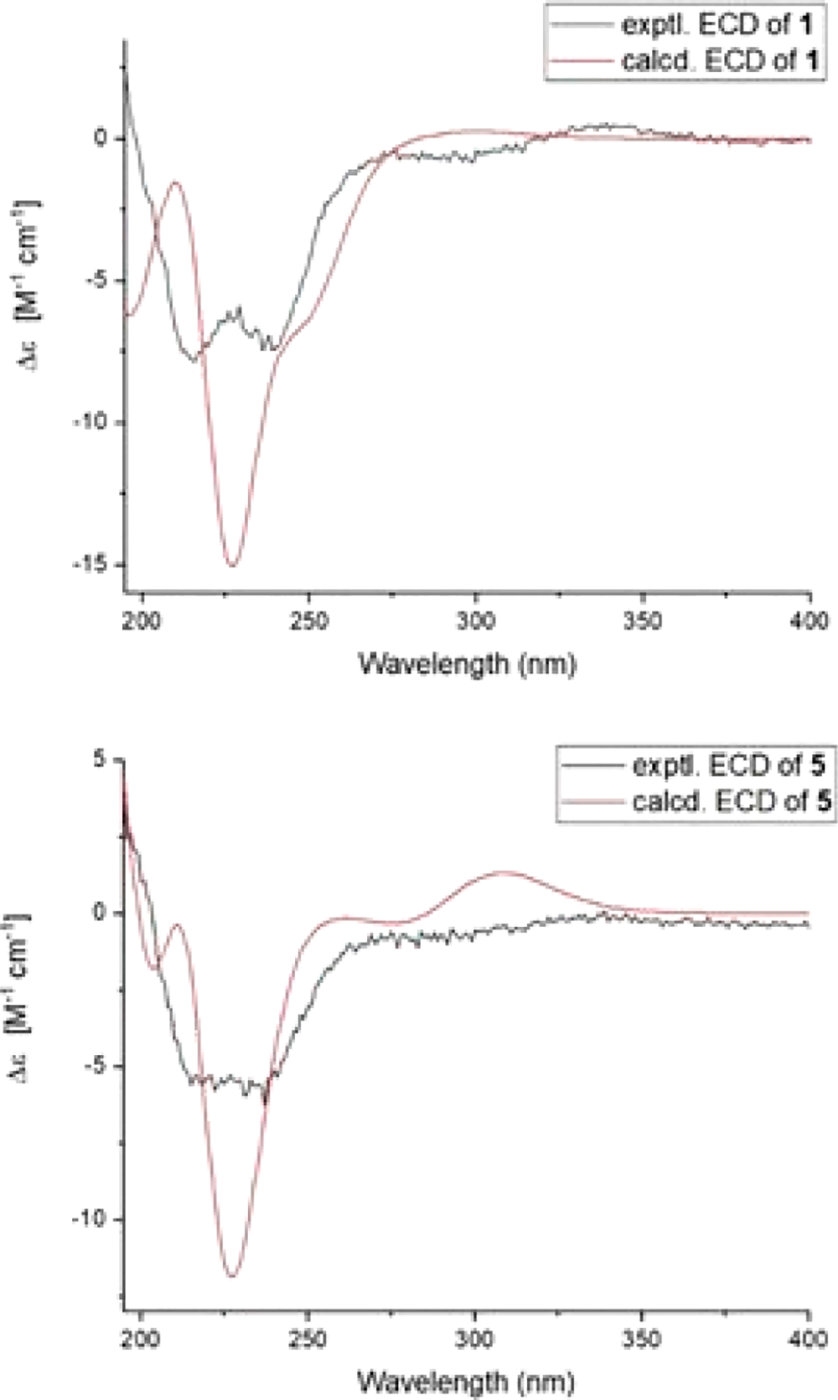
Experimental and calculated ECD spectra of **1** and **5**.

Compound **1** gave a molecular formula of C_22_H_30_O_5_ [HRESIMS *m*/*z* 397.1987 [M + Na]^+^; calcd for C_22_H_30_O_5_Na^+^, 397.1986]. Based on the ^1^H and 2D NMR spectroscopic data (Table 1), the skeleton of **1** was established as a clerodane diterpene with, as for corymbotins A, D, and F (**8**, **3**, and **4**), a Δ^3,4^ double bond [δ_H_ 6.89 (H-3), δ_c_ 151.3 (C-3); δ_c_ 145.9 (C-4)] and a branched six-carbon side chain with a conjugated diene attached to C-9 [δ_H_ 2.00 and 2.19 (H_2_-11), δ_C_ 31.2 (C-11); δ_H_ 5.27 (H-12), δ_C_ 126.2 (C-12); δ_C_ 137.1 (C-13); δ_H_ 6.37 (H-14), δ_C_ 141.3 (C-14); δ_H_ 5.11 and 4.95 (H_2_-15), δ_C_ 111.0 (C-15); and δ_H_ 1.72 (H_3_-16), δ_C_ 12.0 (C-16)]. In addition, the NMR spectra indicated the presence of two formyl groups [δ_H_ 9.40 (H-18), δ_C_ 194.5 (C-18) and δ_H_ 10.00 (H-19), δ_C_ 202.1 (C-19)]. HMBC correlations from H-18 to C-3 (δ_C_ 151.3), C-4 (δ_C_ 145.9), and C-5 (δ_C_ 56.2) and from H-19 to C-5 (δ_C_ 56.2) and C-6 (δ_C_ 73.1) (Figure 2) showed that they are attached to C-4 and C-5, respectively. An HMBC correlation from H-2′ (δ_H_ 2.12) to C-2 (δ_C_ 69.9) and C-1′ (δ_C_ 170.0) confirmed the attachment of an acetoxy moiety at C-2. The relative configuration of **1** was established on the basis of diagnostic NOESY cross-peaks between H-2 and H-10, H-10 and H-19, H-19 and H-11, H-20 and H-1α, H-1β and H-6, H-1β and H-8, and H-6 and H-8. The absolute configuration was determined by a comparison of experimental and calculated ECD spectra of the (2*S*,5*S*,6*S*,8*R*,9*R*,10*S*) stereoisomer (Figure 3). Two negative Cotton effects (CEs) at 216 nm (Δε −7.8) and 239 nm (Δε −7.5) indicated the absolute configuration of **1** as (2*S*,5*S*,6*S*,8*R*,9*R*,10*S*), and the structure of this new compound was thus established as (5*S*,8*R*,9*R*,10*S*)-2*S*-acetoxy-6*S*-hydroxyclerod-3,12,14-trien-18,19-dial.

**Table 1:** ^1^H and ^13^C NMR Spectroscopic Data for Compounds **1** and **5**^a^

	**1**		**5**	
position	δ_C_^b^, type	δ_H_ (*J* in Hz)	δ_C_^b^, type	δ_H_ (*J* in Hz)
1α	25.6, CH_2_	2.22^c^	25.9, CH_2_	2.16, m
1β		1.67, dd (10.4, 2.1)		1.63, m
2	69.9, CH	5.64, ddd (10.5, 6.6, 2.1)	70.2, CH	5.59, ddd (10.5, 6.6, 2.1)
3	151.3, CH	6.89, br s	143.2, CH	6.73, br s
4	145.9, C	-	147.1, C	-
5	56.2, C	-	55.1, C	-
6	73.1, CH	3.96, br dd (11.4, 5.7)	82.6, CH	3.52, m
7	37.4, CH_2_	1.96^c^	32.4, CH_2_	1.76^c^
				2.05, m
8	35.6, CH	1.81, m	35.7, CH	1.77^c^
9	39.5, C	-	39.8, C	-
10	44.4, CH	2.29, dd (13.7, 2.1)	44.5, CH	2.40, dd (14.0, 2.4)
11	31.2, CH_2_	2.00^c^	31.2, CH_2_	1.73^c^
		2.19^c^		2.25, dd (15.9, 8.5)
12	126.2, CH	5.27, br t (6.9)	126.3, CH	5.27, m
13	137.1, C	-	136.7, C	-
14	141.3, CH	6.37, dd (17.2, 10.8)	141.6, CH	6.39, dd (17.4, 10.7)
15	111.0, CH_2_	4.95, d (11.0)	110.7, CH_2_	4.95 d (10.7)
		5.11, d (17.4)		5.09, d (17.4)
16	12.0, CH_3_	1.72, s	12.1, CH_3_	1.71, s
17	15.3, CH3	0.97^c^	15.6, CH_3_	1.01, d (6.1)
18	194.5, CH	9.40, s	190.1, CH	9.30, s
19	202.1, CH	10.00, s	202.0, CH	10.37, s
20	25.7, CH_3_	0.98^c^	25.1, CH_3_	0.90, s
1′	170.0, C	-	170.0, C	-
2′	20.7, CH_3_	2.12, s	20.8, CH_3_	2.10, s
OR-6	-	3.41, br s	57.1, CH_3_	3.32, s

a CDCl_3_; 500.13 MHz for ^1^H and 125.77 MHz for ^13^C NMR; δ in ppm.

b ^13^C NMR data extracted from HSQC and HMBC spectra.

c Overlapping signals.

Compound **5** gave a molecular formula of C_23_H_32_O_5_ (HRESIMS *m*/*z* 411.2145 [M + Na]^+^, calcd for C_23_H_32_O_5_Na^+^, 411.2142). The NMR data were very similar to those of **1**. The major difference was in a methoxy group [δ_H_ 3.32 and δ_C_ 57.1 ppm] that showed an HMBC correlation with C-6 (δ_C_ 82.6) (Figure 2). The relative configuration was established based on NOESY correlations between H-2 and H-10, H-10 and H-19, H-19 and H-6, H-1β and H-6, H-6 and H-8, H-17 and H-11, and H-1α and H-20. The ECD spectrum resembled that of **1**, as it showed two negative Cotton effects at 215 nm (Δε −5.6) and 237 nm (Δε −6.3). The spectrum matched with the calculated ECD spectrum of the (2*S*,5*S*,6*S*,8*R*,9*R*,10*S*) stereoisomer (Figure 3), and the structure of **5** was thus established as (5*S*,8*R*,9*R*,10*S*)-2*S*-acetoxy-6*S*-methoxyclerod-3,12,14-trien-18,19-dial, a new natural product.

A molecular formula of C_27_H_38_O_7_ was calculated for **6** (HRESIMS *m*/*z* 497.2514 [M + Na]^+^, calcd for C_27_H_38_O_7_Na^+^, 497.2510). The 1D and 2D NMR spectroscopic data indicated a structure similar to graveospene H (**2**). However, the acetoxy residue in **2** was replaced by a methacryloxy moiety [δ_C_ 166.7 (C-1′); δ_C_ 136.7 (C-2’); δ_H_ 5.58 and 6.12 (H_2_-3′), δ_C_ 125.2 (C-3′); δ_H_ 1.98 (H_3_-4′), δ_C_ 18.0 (C-4′)], while an HMBC cross-peak between H-2 (δ_H_ 5.51) and C-1′ (Figure 2) indicated the attachment at C-2. NOESY cross-peaks between H-10 and H-20, H-8 and H-20, H-11 and H-17, H-6 and H-8, H-6 and H-19, H-6 and H-18, H-18 and H-19, and H-8 and H-20 were used to establish the relative configuration of the scaffold. The absolute configuration was determined by ECD.^18^ The positive CE at 207 nm (Δε +29.9), together with a negative CE at 232 nm (Δε −9.0) (Figure S27, Supporting Information) indicated a (2*R*,5*S*,6*S*,8*R*,9*R*,10*S*,18*S*,19*S*) configuration. Diterpenoid **6** was thus identified as (2*R*,6*S*,18*S*,19*S*)-2-methylpropenoyloxy-6-hydroxy-18-methoxy-19-acetoxyzuelanin, a new natural product.

The structure of **7** was established with the aid of HRESIMS (*m*/*z* 541.2409 [M + Na]^+^, calcd for C_28_H_38_O_9_Na^+^, 541.2408) and 1D and 2D NMR data (Table 2, Figure 2). The NMR data indicated that it was similar to **3** but with an acetoxy moiety (δ_H_ 2.08, δ_C_ 21.1; δ_C_ 170.1) instead of a hydroxy group at C-6. The relative configuration was determined based on NOESY cross-peaks between H-1α and H-20, H-1β and H-6, H-2 and H-10, H-6 and H-8, H-6 and H-18, H-6 and H-19, H-8 and H-20, and H-11 and H-17. The ECD spectrum showed two negative CEs at 196 (Δε −12.5) and 230 (Δε −12.5) nm (Figure S28, Supporting Information), indicating a (2*S*,5*S*,6*S*,8*R*,9*R*,10*S*,18*S*,19*S*) configuration. Thus, the structure of **7** was established as (2*S*,6*S*,18*S*,19*S*)-tetraacetoxyzuelanin, a new natural product.

**Table 2:** ^1^H and ^13^C NMR Spectroscopic Data for Compounds **6**, **7**, and **9**^a^

	**6**		**7**		**9**	
position	δ_C_^b^, type	δ_H_ (*J* in Hz)	δ_C_, type	δ_H_ (*J* in Hz)	δ_C_, type	δ_H_ (*J* in Hz)
1α	27.0, CH_2_	1.93^c^	26.1, CH_2_	2.21^c^	30.3, CH_2_	1.95^c^
1β		1.93^c^		1.82^c^		1.91^c^
2	66.8, CH	5.51^c^	70.5, CH	5.60, dddd (9.5, 7.0, 2.8, 1.8)	66.1, CH	5.49, br t (4.3)
3	121.5, CH	6.11, dd (4.0, 0.9)	125.2, CH	5.91, br s	121.2, CH	5.93, br d (3.7)
4	146.3, C	-	143.4, C	-	146.0, C	-
5	53.6, C	-	52.0, C	-	52.9, C	-
6	72.9, CH	3.78, dd (12.1, 4.1)	74.8, CH	5.17, dd (12.2, 4.3)	81.9, CH	3.29^c^
7	37.5, CH_2_	1.65^c^	33.4, CH_2_	1.64^c^	31.5, CH_2_	1.46, ddd (12.8, 12.8, 12.8)
		1.72, dd (3.7, 3.7)		1.81^c^		1.88^c^
8	36.7, CH	1.78^c^	36.1, CH	1.94^c^	36.1, CH	1.73^c^
9	37.9, C	-	38.4, C	-	37.7, C	-
10	37.1, CH	2.41, dd (10.1, 7.0)	42.2, CH	2.45, dd (14.0, 2.8)	37.0, CH	2.35, dd (13.1, 4.0)
11	30.4, CH_2_	1.76^c^	29.9, CH_2_	1.72^c^	27.1, CH_2_	1.71^c^
		2.24, dd (16.8, 8.5)		2.25^c^		2.25, dd (16.6, 8.7)
12	129.2, CH	5.40, br dd (7.9, 3.1)	128.5, CH	5.38, br dd (7.6, 2.1)	129.3, CH	5.42, dd (8.2, 2.4)
13	135.5, C	-	135.8, C	-	135.3, C	-
14	141.3, CH	6.26, dd (17.4, 10.7)	141.1, CH	6.32, dd (17.2, 10.8)	141.3, CH	6.31, dd (17.2, 10.8)
15	110.7, CH_2_	4.92 d (10.7)	111.0, CH_2_	4.95, d (10.7)	110.7, CH_2_	4.94, d (10.7)
		5.09, d (17.4)		5.11, d (17.4)		5.10, d (17.1)
16	11.8, CH_3_	1.67^c^	11.8, CH_3_	1.68^c^	11.8, CH_3_	1.68, s
17	15.5, CH_3_	0.94, d (6.7)	15.3, CH_3_	0.94, d (6.7)	15.7, CH_3_	0.97, d (7.0)
18	104.6, CH	5.53^c^	94.8, CH	6.49, dd (1.5, 1.5)	96.2, CH	6.67, dd (1.5, 1.2)
19	96.7, CH	6.49, s	97.0, CH	6.54, s	97.7, CH	6.50, s
20	24.9, CH_3_	0.81, s	24.9, CH_3_	0.87, s	24.9, CH_3_	0.83, s
1′	166.7, C	-	169.7, C	-	173.8, C	-
2′	136.7, C	-	21.1, CH_3_	2.08^c^	27.9, CH_2_	2.42, qd (7.6, 1.2)
3′	125.2, CH_2_	5.58, dq (1.7, 1.4)	170.1, C	-	9.1, CH_3_	1.21, dd (7.6, 7.6)
		6.12, dq (1.7, 0.9)				
4′	18.0, CH_3_	1.98, dd (1.4, 0.9)	21.1, CH_3_	2.08^c^	-	-
OR-6	-	-	170.7, C	-	57.4, CH_3_	3.31, s
OR1″-18	55.6, CH_3_	3.41, s	21.1, CH_3_	2.08^c^	170.1, C	-
OR2″-18	-	-	169.3, C	-	21.2, CH_3_	2.10, s
OR1″′-19	169.6, C	-	21.1, CH_3_	1.96, s	169.5, C	-
OR2″′-19	21.4, CH_3_	1.92, s	26.1, CH_2_	2.21^c^	21.6, CH_3_	1.95, s
				1.82^c^		

a CDCl_3_; 500.13 MHz for ^1^H and 125.77 MHz for ^13^C NMR; δ in ppm.

b ^13^C NMR data extracted from HSQC and HMBC spectra.

c Overlapping signals.

Compound **9** (HRESIMS *m*/*z* 527.2618 [M + Na]^+^, calcd for C_28_H_40_O_8_Na^+^, 527.2616) gave a molecular formula of C_28_H_40_O_8_. 1D and 2D NMR data (Table 2, Figure 2) closely resembled those of **8**, the only difference being in the presence of a propylate residue at C-2 [δ_C_ 173.8 (C-1′); δ_H_ 2.42 (H_2_-3′), δ_C_ 27.9 (C-2′); δ_H_ 1.21 (H_3_-3′), δ_C_ 9.1 (C-3′)]. NOESY correlations between H-6 and H-8, H-8 and H-20, H-10 and H-20, H_3_CO-6 and H-19, H_3_CO-6 and H-18, and H-18 and H-19 established the relative configuration, and CEs at 202 (Δε +26.19) and 234 (Δε −8.3) nm in the ECD spectrum (Figure S28, Supporting Information) confirmed the absolute configuration as 2*R*,5*S*,6*S*,8*R*,9*R*,18*R*,19*S*. The structure of **9** was thus established as (2*R*)-propanoyloxy-(6*R*)-methoxy-18*R*,19*S*-diacetoxyzuelanin, a new natural product.

**Figure 4:**
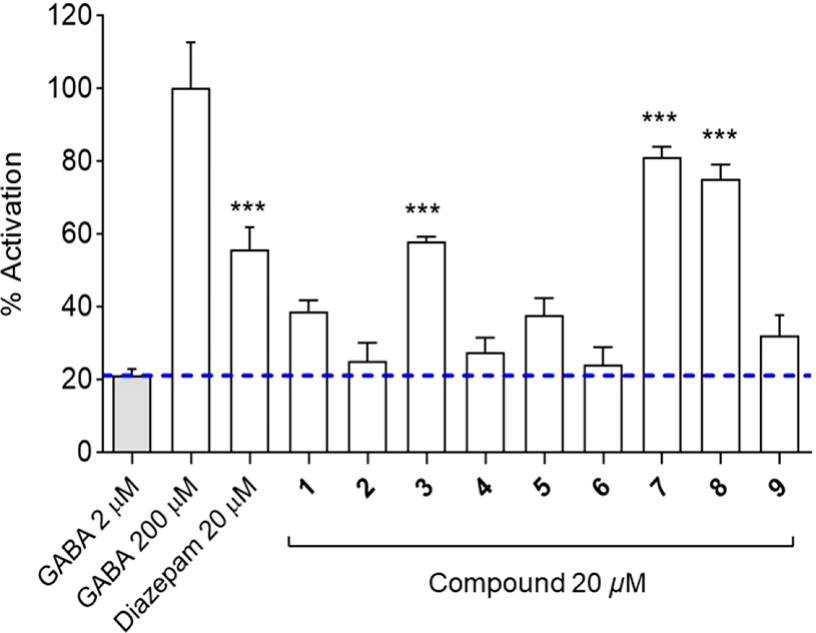
Percentage of activation for compounds **1**–**9** (in the presence of 2 μM GABA), along with 2 μM GABA (control), 200 μM GABA (100%), and 20 μM diazepam (in the presence of 2 μM GABA) (*n* = 4, means ± SEM). The final DMSO concentration in the assay was 0.1%. The *** above the bars indicate statistical significance with *p* ≤ 0.001.

**Figure 5:**
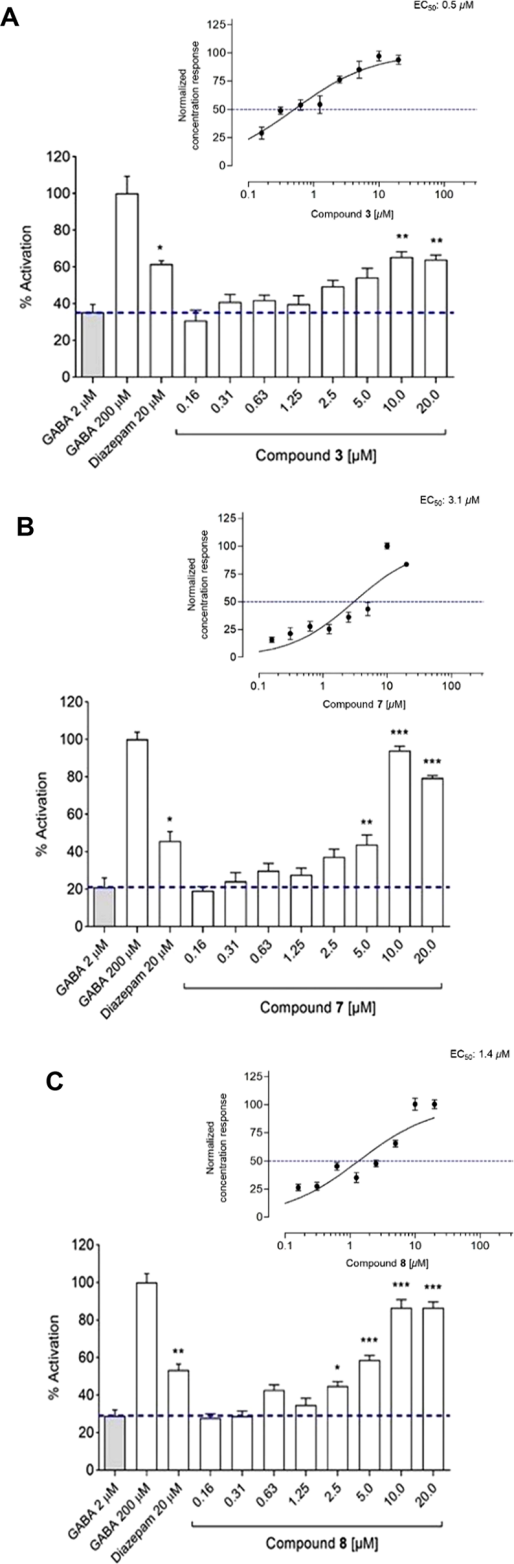
Percentage of activation for compounds **3** (A), **7** (B), and **8** (C) (in the presence of 2 μM GABA), along with 2 μM GABA (control), 200 μM GABA (100%), and 20 μM diazepam (in the presence of 2 μM GABA) (*n* = 10, means ± SEM). The plots above each bar graph show the corresponding concentration–response curves with calculated EC_50_ values. The final DMSO concentration in the assay was 0.1%. The *, **, and *** above the bars indicate statistical significance with *p* ≤ 0.05, *p* ≤ 0.01, and *p* ≤ 0.001, respectively.

Compounds **1**–**9** were first tested at a single concentration of 20 μM in the FLIPR assay (Figure 4). The results showed that **3**, **7**, and **8** significantly enhanced the GABA signals. Diterpenoids **1** and **5** were found to have moderate activity (% activation ∼40%), while **2**, **4**, **6**, and **9** displayed no discernible activity. Concentration–response and EC_50_ values were thus determined for compounds **3**, **7**, and **8**. They displayed significant positive allosteric modulation of the GABA signals, with EC_50_ values of 0.5, 4.6, and 1.4 μM, respectively (Figure 5). When compared to other known allosteric GABA_A_ receptor modulators previously tested in the FLIPR assay, such as piperine (EC_50_: 5.8 μM), magnolol (EC_50_: 4.8 μM), and valerenic acid (EC_50_: 12.6 μM),^14^ diterpenoids **3**, **7**, and **8** were more potent.

GABA_A_ receptors possess several binding sites.^5,6^ The binding sites of most classical orthosteric and allosteric agonists and antagonists have been shown to be located in the extracellular domain. In addition, the interfaces between subunits of GABA_A_ receptors in the transmembrane domain (TMD) contain specific binding sites for ligands of different types including lipids and neurosteroids, while direct channel blockers bind within the ion pore formed by the GABA_A_ receptor pentamer.^22−25^

We investigated the possible binding site of diterpenoid **8** utilizing known receptor agonists/antagonists for the different allosteric binding sites. In a first step, the assay conditions for the binding sites and agonist/antagonist combinations were optimized. The potentiation of the GABA signal by diazepam (2 μM) was abolished by flumazenil (0.001–100 μM) in a concentration-dependent manner (Figure S29, Supporting Information), and activation by the neurosteroid allopregnanolone (0.5 μM) was abrogated by increasing concentrations of pregnenolone sulfate (PREGS, 0.001–100 μM, Figure S30, Supporting Information). The barbiturate-binding site was validated with the aid of the positive modulator etazolate (Figure S31, Supporting Information), since no antagonists of this binding site are known. In contrast, no activation was seen in the presence of ethanol at concentrations up to 640 mM (Figure S32, Supporting Information). This is in line with reports that GABA_A_ receptors containing γ_2_ subunits are only weakly sensitive to ethanol.^23,24^

To assess a possible interaction with the benzodiazepine binding site, compound **8** (5 μM) was tested together with increasing concentrations of the antagonist flumazenil (0.001–10 μM) (Figure S33A, Supporting Information). The activation by **8** was not abrogated even at the highest flumazenil concentration. When combining increasing concentrations of compound **8** (0.5–8 μM) with diazepam (2 μM) an additive effect was observed (Figure S33B, Supporting Information). These data indicated that the diterpenoid was interacting with a binding site that was independent of the benzodiazepine site. The potentiation of the GABA signal by etazolate (2 μM) was further increased by increasing concentrations of **8** (0.5–8 μM) (Figure S33, Supporting Information), suggesting that the diterpenoid interacted with an allosteric site other than the barbiturate-binding site of the GABA_A_ receptor. Given that no antagonists at this binding site are currently known, no antagonist experiments were conducted. Neurosteroids and general anesthetics are known to bind on sites located in the TMD and subunit interface of the GABA_A_ receptor.^25−27^ To assess a possible interaction with an antagonist neurosteroid binding site, diterpenoid **8** (10 μM) was tested in combination with the negative allosteric modulator pregnenolone sulfate (PREGS) and the positive allosteric modulator allopregnanolone (Figure S35, Supporting Information). At lower concentrations, PREGS had no effect, and a decrease of the GABA signal was only seen at the highest concentration of PREGS (10 μM). When **8** (0.5–4.0 μM) was tested together with the positive allosteric modulator allopregnanolone (0.25 μM), the activation increased in a concentration-dependent manner indicating additive effects of **8** and allopregnanolone.

**Figure 6:**
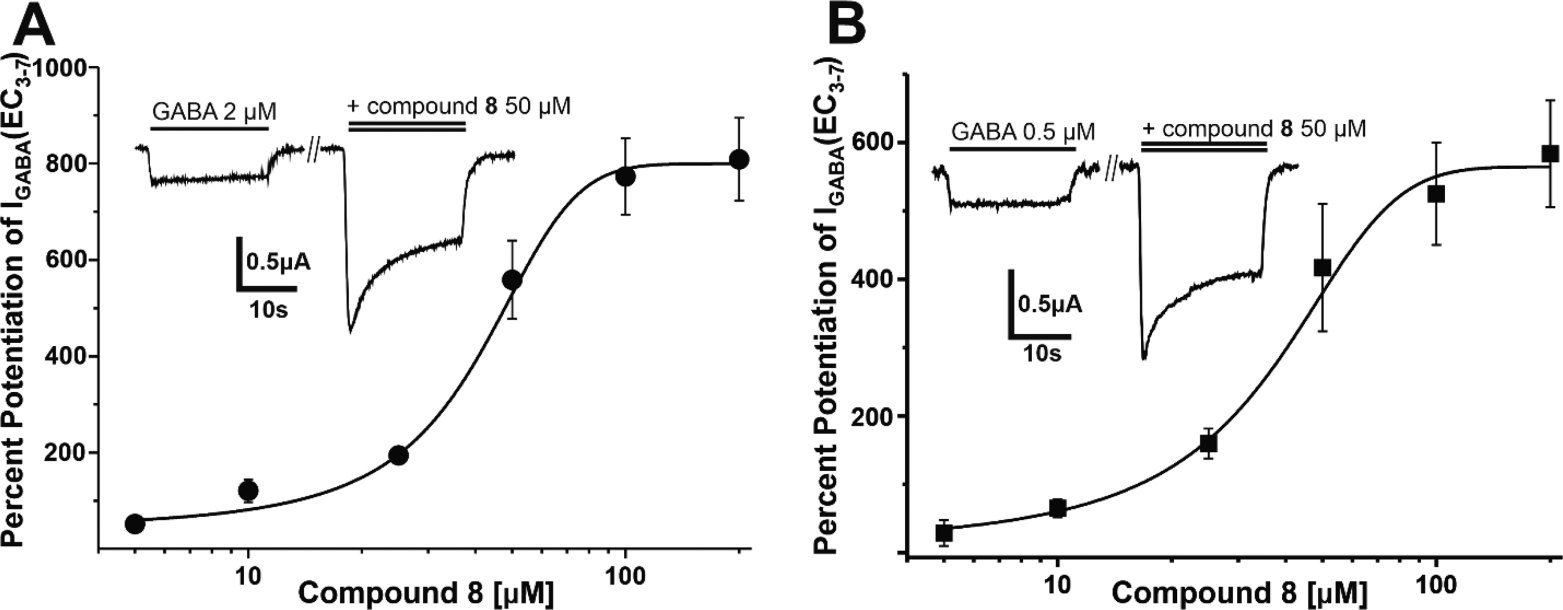
Concentration–effect curves for the enhancement of *I*_GABA_ through GABA_A_ receptors composed of α_1_β_2_γ_2S_ (A) and α_1_β_2_ (B) subunit compositions in *Xenopus* oocytes, by increasing concentrations of **8** in the presence of GABA EC_3–7_. Representative traces for the enhancement of *I*_GABA_ for both subunit compositions by 50 μM of **8** (double bar indicates coapplication of GABA and **8**) are depicted in the inset. Data points represent means ± SEM recorded with at least three oocytes.

Finally, the GABAergic activity of **8** was validated in voltage clamp studies on *Xenopus laevis* oocytes transiently expressing GABA_A_ receptors of the α_1_β_2_γ_2_S and α_1_β_2_ subtypes, respectively. In the presence of GABA EC_3–7_, compound **8** potentiated GABA-induced chloride currents (*I*_GABA_) with both receptor subunit compositions (EC_50_ (α_1_β_2_γ_2S_) = 43.6 ± 11.4 μM; *E*_max_ = 809 ± 86% (*n* = 3) and EC_50_ (α_1_β_2_) = 57.6 ± 23.4 μM; *E*_max_ = 534 ± 88% (*n* = 3); Figure 6). *I*_GABA_ stimulation by compound **8** (50 μM) was not dependent on a γ subunit and not prevented by flumazenil (2 μM), thereby indicating an interaction independent of the benzodiazepine binding site.

Cryoelectron microscopic structures of GABA_A_ receptors have been recently published^28−30^ and revealed the existence of separate binding sites for agonistic and antagonistic neurosteroids on the α_1_ subunit and at the interface of the β_3_/α_1_ subunits.^25,31^ The inhibition of the GABA signal at the highest PREGS concentration (10 μM, Figure S35, Supporting Information) is more likely to reflect a direct inhibition of *I*_GABA_ than interaction with a binding site for neurosteroids.^32,33^ The additive effects of the positive allosteric modulator allopregnanolone and **8** suggest an independent action of these ligands.

Taken together, the lack of flumazenil action on the stimulation of α_1_β_2_γ_2S_ receptors by **8**, the additive effects of saturating concentrations of **8** with diazepam, the barbiturate etazolate, the agonistic neurosteroid allopregnanolone, and the lack of effects of PREGS at concentrations <10 μM suggest a separate binding site for compound **8** on GABA_A_ receptors. While the EC_50_ values in *Xenopus* oocytes expressing α_1_β_2_γ_2S_ and α_1_β_2_ receptors are relatively moderate in potency, the maximal potentiations (*E*_max_) of 809 and 549%, respectively, are notable and significantly higher than that of drugs such as diazepam (∼200% in the *Xenopus* oocyte assay).^34^ However, further investigations are needed to better understand if **8** binds to a novel binding pocket or if its action is linked to one of the other known putative binding sites of GABA_A_ receptor modulators.^5^

## Experimental Section

### General Experimental Procedures

Optical rotations were measured in chloroform on a P-2000 digital polarimeter (JASCO) equipped with a sodium lamp (589 nm) and a 10 cm temperature-controlled microcell. Electronic circular dichroism (ECD) spectra were recorded on a Chirascan CD spectrometer with 1 mm path precision cells (110 QS, Hellma Analytics), at a concentration of 0.1–0.2 mg/mL in acetonitrile (CH_3_CN). NMR spectra were measured with a Bruker Avance III spectrometer operating at 500.13 MHz for ^1^H and 125.77 MHz for ^13^C. ^1^H NMR, COSY, HSQC, HMBC, and NOESY spectra were measured with a 1 mm TXI probe at 23 °C. ^13^C NMR spectra were obtained in 3 or 5 mm tubes with a BBO probe at 23 °C. CDCl_3_ for NMR was from Armar Chemicals (Döttingen).

HPLC-PDA-ELSD-ESIMS analysis was performed with an instrument consisting of a degasser, quaternary pump (LC-20AD), column oven (CTO-20AC), PDA detector (SPD-M20A), and triple quadrupole mass spectrometer (LCMS-8030) (all Shimadzu), connected via a T-split to an ELSD 3300 detector (Alltech). A SunFire C_18_ column (3.5 μm, 150 × 3.0 mm i.d., with a guard column of 10 mm × 3.0 mm i.d.; Waters) was used. Data acquisition and processing were performed with Lab Solution software (Shimadzu). Time-based microfractionation into 96-deep-well plates was carried out with the analytical HPLC system. Instead of the mass spectrometer, an FC 204 fraction collector (Gilson) was connected.

Semipreparative HPLC was carried out with an HP 1100 Series instrument consisting of a quaternary pump, autosampler, column oven, and a G13115B diode array detector (all Agilent). A SunFire Prep C_18_ column (5 μm, 10 × 150 mm) equipped with a guard column (10 × 10 mm) (Waters) and, for the normal phase, a Nucleodur Prep 100-5 CN column (5 μm, 10 × 150 mm) equipped with a guard column (10 × 10 mm) (Macherey-Nagel) were used for separation.

HPLC-grade acetonitrile (Scharlau Chemie) and water from a Milli-Q water purification system (Merck Millipore) with 0.1% formic acid were used for reversed phase separations. Extra pure *n*-heptane (Scharlau) and HPLC-grade 2-propanol (Macron Fine Chemicals) were used for the normal phase (semipreparative CN column). Solvents used for extraction and column chromatography were of technical grade (Romil Pure Chemistry) and were redistilled before use. Silica gel (0.063–0.200 μm, Merck) was used for flash column chromatography on a Puriflash 4100 system (Interchim). HRESIMS data were measured on a LQT XL Orbitrap mass spectrometer (Thermo Scientific) via direct injection. Evaporation of microfractions was done with a EZ-2 plus vacuum centrifuge (Genevac).

### Plant Material

Leaves of *Casearia corymbosa* were collected by Alex Espinosa in El Cope, 5 km from Ilegar al Pablado, Panama. The material was authenticated by Alex Espinosa, taxonomist at CIFLORPAN, and voucher specimens have been deposited at the Herbarium of the University of Panama (PMA), Panama (voucher number 7166), and at the Division of Pharmaceutical Biology, University of Basel, Switzerland (voucher # 853).

### Microfractionation for Activity Profiling

The *C. corymbosa* EtOAc extract from the in-house extract library^19^ was separated by analytical RP-HPLC [0.1% aqueous formic acid (A), 0.1% formic acid in CH_3_CN (B), 0–30 min (5–100% B); 30–37 min (100% B); flow rate 0.5 mL/min; injection volume 3 × 40 μL of a solution of 10 mg/mL in DMSO. From *t*_R_ 8 to 32 min, fractions of 3 min each were collected into a 96-deep-well plate with a conical bottom (Biotage) and dried for 12 h at 37 °C in a Genevac EZ-2 vacuum evaporator. The residues were redissolved prior to the bioassay.

### Extraction and Isolation

Powdered *C. corymbosa* leaves (60 g) were macerated at r.t. under stirring with 3 × 300 mL EtOAc for 2 days each, to afford 5.8 g of crude extract. The extract was separated on a silica gel column (49 × 460 mm) using a step gradient (*n*-hexane, *n*-hexane/EtOAc, EtOAc, EtOAc/MeOH, and MeOH). The flow rate was 30 mL/min, and 20 fractions were collected. Fractions were analyzed by HPLC-PDA-ESIMS, and compounds detected in the active time windows of the HPLC activity profile were localized in fractions 7 to 16. Fractions 8 (51 mg) and 9 (110 mg) were submitted to semipreparative RP-HPLC [H_2_O + 0.1% formic acid (A), CH_3_CN + 0.1% formic acid (B); isocratic 64% B (1–30 min); flow rate 4.0 mL/min]. Fractions 8 and 9 yielded, respectively, eight (A1–A8) and seven (B1–B7) subfractions. Subfractions of interest were separated by semipreparative HPLC on a CN column with isopropyl alcohol (A) and heptane (B) as mobile phases. Fraction A5 (10 mg) [isocratic 99% B (1–30 min); flow rate 4.0 mL/min] afforded compound **8** (2.1 mg, *t*_R_ 26.3 min). Fraction A6 (10 mg) [isocratic 97% B (1–30 min); flow rate 3.0 mL/min] gave compound **9** (0.3 mg, *t*_R_ 24.5 min). Fraction B1 (10 mg) [isocratic 95% B (1–30 min); flow rate 3.0 mL/min] yielded compound **3** (0.3 mg, *t*_R_ 21.6 min). Fraction B3 (10 mg) [isocratic 97% B (1–30 min); flow rate 3.0 mL/min] afforded compound **7** (0.6 mg, *t*_R_ 18.2 min). Fraction B4 (60 mg) [isocratic 97% B (1–30 min); flow rate 3.0 mL/min] afforded compounds **5** (0.6 mg, *t*_R_ 17.2 min) and **8** (4 mg, *t*_R_ 23.1 min). Fraction 10 (800 mg) was submitted to preparative RP-HPLC [H_2_O + 0.1% formic acid (A), MeOH + 0.1% formic acid (B); isocratic 75% B (1–30 min); flow rate 20 mL/min] to afford two fractions (C1 and C2). Fraction C1 (80 mg) was submitted to semipreparative RP-HPLC [H_2_O + 0.1% formic acid (A), CH_3_CN + 0.1% formic acid (B); isocratic 54% B (1–30 min); flow rate 4.0 mL/min] to yield seven subfractions (C1a–C1g). Fractions C1d (6.1 mg) and C1e (12.6 mg) were separated by semipreparative HPLC on a CN column with 2-propanol (A) and *n*-heptane (B) as mobile phases. Fraction C1d [isocratic 97% B (1–30 min); flow rate 3.0 mL/min] afforded compound **2** (1.6 mg, *t*_*R*_ 13.6 min) and compound **1** (1.0 mg, *t*_R_ 5.2 min). Fraction C1e [isocratic 96% B (1–30 min); flow rate 3.0 mL/min] afforded compound **3** (6.0 mg, *t*_R_ 23.3 min) and compound **1** (0.8 mg, *t*_R_ 6.4 min). Fraction C2 (100 mg) was submitted to semipreparative HPLC on a CN column [2-propanol (A), *n*-heptane (B), isocratic 97% B (1–35 min); flow rate 3.0 mL/min] to obtain compounds **6** (2.5 mg, *t*_R_ 14.2 min) and **4** (15 mg, *t*_R_ 31.4 min). The purity of compounds was established by HPLC as ≥95%.

#### (5*S*,8*R*,9*R*,10*S*)-2*S*-Acetoxy-6*S*-hydroxyclerod-3,12,14-trien-18,19-dial (**1**)

[α]_D_^25^ −59.1 (*c* 0.02, CH_2_Cl_2_); ECD (CH_3_CN) 216 (Δε −7.8), 239 (Δε −7.5), 299 (Δε – 0.9) nm; ^13^C NMR and ^1^H NMR data, see Table 1; HRESIMS *m*/*z* 397.1987 [M + Na]^+^ (calcd for C_22_H_30_O_5_Na^+^, 397.1986).

#### Graveospene H (**2**)

[α]_D_^25^ −48.0 (*c* 0.03, CH_2_Cl_2_); ECD (CH_3_CN) 206 (Δε +0.1), 228 (Δε −11.1) nm; ^13^C NMR and ^1^H NMR data, see Table S1, Supporting Information; HRESIMS *m*/*z* 471.2355 [M + Na]^+^ (calcd for C_25_H_36_O_7_Na^+^, 471.2354).

#### Corymbotin D (**3**)

[α]_*D*_^25^ −31.4 (c 0.04, CH_2_Cl_2_); ECD (CH_3_CN) 207 (Δε +1.6), 231 (Δε −10.0) nm; ^13^C NMR and ^1^H NMR data, see Table S1, Supporting Information; HRESIMS *m*/*z* 499.2303 [M + Na]^+^ (calcd for C_26_H_36_O_8_Na^+^, 499.2303).

#### Corymbotin F (**4**)

[α]_D_^25^ +57.1 (*c* 0.02, CH_2_Cl_2_); ECD (CH_3_CN) 207 (Δε +31.9), 231 (Δε −10.1) nm; ^13^C NMR and ^1^H NMR data, see Table S2, Supporting Information; HRESIMS *m*/*z* 525.2458 [M + Na]^+^ (calcd for C_28_H_38_O_8_Na^+^, 525.2459).

#### (5*S*,8*R*,9*R*,10*S*)-2*S*-Acetoxy-6*S*-methoxyclerod-3,12,14-trien-18,19-dial (**5**)

[α]_D_^25^ −62.5 (*c* 0.01, CH_2_Cl_2_); ECD (CH_3_CN) 215 (Δε −5.6), 237 (Δε −6.3), 299 (Δε −0.9) nm; ^13^C NMR and ^1^H NMR data, see Table 1; HRESIMS *m*/*z* 411.2145 [M + Na]^+^ (calcd for C_23_H_32_O_5_Na^+^, 411.2142).

#### (2*R*)-2-Methylpropenoyloxy-6*S*-hydroxy-18*S*-methoxy-19*S*-acetoxyzuelanin (**6**)

[α]_D_^25^ +57.9 (*c* 0.02, CH_2_Cl_2_); ECD (CH_3_CN) 207 (Δε +29.9), 232 (Δε −9.0) nm; ^13^C NMR and ^1^H NMR data, see Table 2; HRESIMS *m*/*z* 497.2514 [M + Na]^+^ (calcd for C_27_H_38_O_7_Na^+^, 497.2510).

#### (2*S*,6*S*,18*S*,19*S*)-Tetraacetoxyzuelanin (**7**)

[α]_D_^25^ −51.5 (*c* 0.03, CH_2_Cl_2_); ECD (CH_3_CN) 196 (Δε −12.5), 230 (Δε −12.5) nm; ^13^C NMR and ^1^H NMR data, see Table 2; HRESIMS *m*/*z* 541.2409 [M + Na]^+^ (calcd for C_28_H_38_O_9_Na^+^, 541.2408).

#### Corimbotin A (**8**)

[α]_D_^25^ −44.2 (*c* 0.05, CH_2_Cl_2_); ECD (CH_3_CN) 204 (Δε +2.8), 232 (Δε −10.3) nm; ^13^C NMR and ^1^H NMR data, see Table S2, Supporting Information; HRESIMS *m*/*z* 513.2460 [M + Na]^+^ (calcd for C_27_H_38_O_8_Na^+^, 513.2459).

#### (2*R*)-Propanoyloxy-(6*R*)-methoxy-18*R*,19*S*-diacetoxyzuelanin (**9**)

[α]_D_^25^ +53.8 (*c* 0.01, CH_2_Cl_2_); ECD (CH_3_CN) 202 (Δε +26.1), 234 (Δε −8.3) nm; ^13^C NMR and ^1^H NMR data, see Table 2; HRESIMS *m*/*z* 527.2618 [M + Na]^+^ (calcd for C_28_H_40_O_8_Na^+^, 527.2616).

### Computational Methods

Conformational analysis was performed with Schrödinger MacroModel 9.8 (Schrödinger, LLC, New York, USA) employing the OPLS2005 (optimized potential for liquid simulations) force field in water for ECD calculations. The five conformers with the lowest energy were selected for geometrical optimization and energy calculation by applying DFT with the Becke’s nonlocal three parameter exchange and correlation functional and the Lee–Yang–Parr correlation functional level (CAM-B3LYP), using the 6-31G(d,p) basis set and the SCRF method with the CPMC model for solvation (MeOH) with the Gaussian 09 program package.^35^ Excitation energy (denoted by wavelength in nm), rotator strength (Rstr), dipole velocity (Rvel), and dipole length (Rlen) were calculated in CH_3_CN by TD-DFT/CAM-B3LYP/6-31G(d,p). ECD curves were obtained on the basis of rotator strengths with a half-band of 0.3 eV using SpecDis v1.71.^36^ ECD spectra were calculated from the spectra of individual conformers according to their contribution calculated by Boltzmann weighting.

### FLIPR Assay

Chinese hamster ovary (CHO) cells stably expressing the GABA_A_ receptor with the α_1_β_2_γ_2_ subunit composition were passaged following an established protocol.^15,16^ The Chinese hamster ovary (CHO) cell line stably expressing the α_1_β_2_γ_2_ GABA_A_ receptor subtype was cloned by B’SYS. Cells were cultured in a DMEM F-12 nutrient mixture supplemented with 10% FBS and 1% penicillin/streptomycin, under antibiotic pressure with 200 μg/mL of hygromycin B, 5 μg/mL of puromycin, and 100 μg/mL of zeocin. Cells were incubated in humidified air at 37 °C and 5% CO_2_, until 80–90% cell confluency. The passage numbers used in this study were from 10 to 30. Briefly, cells of ca. 80–90% confluency were seeded in a 96-well black-walled plate at a density of 60 000 cells/well in 100 μL of culture medium. The plate was incubated in humidified air at 37 °C and 5% CO_2_ for 24 h to allow cells to adhere. Then, 100 μL of FLIPR red dye solution (10.4 mL of assay buffer and one vial of red FLIPR assay reagent) was added to each well. The plate was further incubated for 30 min to allow the dye to penetrate into the cell membrane (assay plate). During the incubation time, a compound plate (clear 96-well plate) with controls, extracts, microfractions, compounds, and blanks was prepared (200 μL/well). Test solutions of extracts, microfractions, and compounds were prepared in buffer (consisting of 10% HBSS and 2% HEPES in sterilized H_2_O) and DMSO.

For the extract library screening, 2 μL of a 10 mg/mL DMSO stock solution of the extracts was added to 198 μL of buffer and transferred into the compound plate. The final test concentration for extracts was 20 μg/mL, with a final DMSO concentration in the assay of 0.1%. For extracts tested, active (>35% potentiation) serial dilutions were prepared from the stock solution to obtain final test concentrations of 0.16, 0.31, 0.63, 1.25, 2.50, 5.00, 10.00, and 20.00 μg/mL.

For the testing of microfractions, 10 μL of DMSO was added to each well of the 96-well deepwell plate, followed by 990 μL of HBSS buffer. The plate was shaken for 10 min at 500 rpm on a MixMate plate shaker (Eppendorf). Next, 100 μL of each well was mixed in Eppendorf tubes with 100 μL o HBSS buffer (1:1 dilution), and the solutions were transferred into the compound plate. Aliquots of 50 μL were transferred from the compound plate to the assay plate during the fluorescent measurement.

To test the pure compounds, stock solutions of 20 mM in DMSO were prepared. Compounds were assayed at a final concentration range of 0.16 to 20.00 μM (8× dilution series) or alternatively from 0.01 to 20.00 μM (12× dilution series). Aliquots of 200 μL from each dilution were transferred into wells of the compound plate and tested.

A series of controls was included in each assay. GABA at 200 μM was used to represent 100% activation of the receptor, while GABA at 2 μM represented the minimum activation (EC_10_). The positive control was diazepam at 20 μM in the presence of GABA at 2 μM, and buffer with 0.1% DMSO was used as a blank. GABA solutions (2 and 200 μM) were prepared from a 100 mM stock solution. In order to achieve a final test concentration of 20 μM diazepam in the assay plate, a 100 μM working solution was prepared from a stock solution (1 mg/mL in MeOH).

All test solutions were placed in the appropriate position in the compound plate (200 μL). Right after the final incubation period of the assay plate, the compound and assay plates were placed into a FlexStation 3 (Molecular Devices). The FlexStation parameters used in this study were as previously published.^14^ Fluorescence was recorded for 500 s. For the first 25 s, the background fluorescence of the assay plate was measured (background signal). At 25 s, 50 μL of the test solutions (dilutions from compounds, extracts, microfractions, diazepam, and blanks) was transferred from the compound plate into the assay plate. The fluorescence was then recorded until 295 s. At 295 s, 25 μL of the GABA solutions (final concentration of 2 or 200 μM) was transferred into each well of the assay plate, and fluorescence was recorded until 500 s. The change in fluorescence intensity measured between 270 and 330 s was used for the calculation of percentage activation.

### Investigation of Allosteric Binding Sites

For the benzodiazepine binding site, a serial dilution of flumazenil (0.001 to 100 μM, 100 mM stock solution in DMSO) with diazepam at a fixed concentration of 2 μM was prepared in the compound plate and tested in the FLIPR assay according to the above protocol, with the addition of 2 μM GABA at 295 s. Then, compound **8** at a concentration of 5 μM was tested with increasing concentrations of flumazenil (0.001 to 10 μM). For an additive potentiation, increasing concentrations of compound **8** (0.5, 1.0, 2.0, 4.0, and 8.0 μM, from a 16 mM stock solution in DMSO) were tested together with diazepam at a fixed concentration of 2 μM.

Titrations of etazolate (0.78 to 25 μM, prepared from a 25 mM stock solution) were used to evaluate the barbiturate-binding site. For an additive potentiation, increasing concentrations of compound **8** (0.5 to 8.0 μM) were tested together with a fixed concentration of etazolate at 0.78 μM.

For the neurosteroid binding site, a serial dilution of PREGS (0.001 to 100.0 μM, prepared from a 100 mM stock solution) was tested together with allopregnanolone at a fixed concentration of 0.5 μM. Then, compound **8** at a fixed concentration of 10 μM was tested in the presence of increasing concentrations of PREGS (0.001 to 10 μM). For an additive potentiation, increasing concentrations of compound **8** (0.5, 1.0, 2.0, and 4.0 μM) were tested in the presence of a fixed concentration of 0.25 μM allopregnanolone. For all binding site experiments, 2 μM GABA was added at 295 s. In addition, controls of 2 μM GABA, 200 μM GABA, and 0.2% DMSO were used in each assay plate.

### Statistical Analysis

GraphPad Prism version 5 (GraphPad Software) was used for the calculations and graphical plots. Grubbsʼ test of the GraphPad outlier calculator was used (α = 0.05) to determine data outliers. After removal of outliers, percentage activation (%) was calculated by normalizing the readouts of the controls and test samples with that of 200 μM GABA. To compute the statistical significance, the average total activation of each test sample was compared to the EC_10_ (2 μM GABA) by one-way analysis of variance, followed by Dunnettʼs multiple comparison test. The statistical significance indicated with *, **, ***, and ****, respectively, represents *p* ≤ 0.05, *p* ≤ 0.01, *p* ≤ 0.001, and *p* ≤ 0.0001.

### Two-Microelectrode Voltage Clamp Assay with *Xenopus laevis* Oocytes

Recombinant GABA_A_ receptors (α_1_β_2_γ_2_s and α_1_β_2_) were expressed in *X. laevis* oocytes by cRNA injection, as previously described.^37^ Two-microelectrode voltage clamp measurements were performed between days 1 and 5 after injection of cRNA of the respective subunits, using a TURBO TEC 03X amplifier (npi Electronic) at a holding potential of −70 mV and pCLAMP 10 data acquisition software (Molecular Devices). The bath solution contained 90 mM NaCl, 1 mM KCl, 1 mM MgCl_2_, 1 mM CaCl_2_, and 5 mM HEPES (pH 7.4), and the electrode filling solution contained 2 M KCl. Test solutions (100 μL) were applied to the oocytes at a speed of 300 μL/sec by means of the ScreeningTool (npi electronic) automated fast perfusion system. GABA EC_3–7_ was determined through a concentration–response experiment with GABA. A stock solution of compound **8** (100 mM) in DMSO was diluted with a bath solution containing GABA EC_3–7_ to obtain appropriate working solutions according to a validated protocol. Enhancement of the *I*_GABA_ was defined as (*I*_(GABA+Comp)_/*I*_GABA_) – 1, where *I*_(GABA+Comp)_ is the current response in the presence of a given compound, and *I*_GABA_ is the control of the GABA-induced chloride current. *E*_max_ reflects the maximal *I*_GABA_ enhancement. Concentration–response curves were generated, and the data were fitted by nonlinear regression analysis using Origin Software (OriginLab Corporation, USA) and are given as the means ± SEM of at least three oocytes from ≥2 batches. Diazepam (Sigma) and flumazenil (Sigma-Aldrich) were used as positive controls at 2 μM.

## Supporting Information Available

NMR spectra and tables, ECD spectra of compounds, testing of extract, control experiments for various binding sites in the transfected CHO cells, and testing with different agonists/antagonists (PDF)

## Notes

^+^ Mahabir P. Gupta passed away on December 14, 2020.
