# Supplementary material for: Clerodane Diterpenes from Casearia corymbosa as Allosteric GABAA Receptor Modulators
Source: J Nat Prod. 2022 Apr 27;85(5):1201–10. doi: 10.1021/acs.jnatprod.1c00840 (PMC9150179; doi:10.1021/acs.jnatprod.1c00840)
Supplement: Supplementary file 1 — np1c00840_si_001.pdf [file np1c00840_si_001.pdf]

Supporting Information

Clerodane Diterpenes from *Casearia corymbosa* as Allosteric GABA$_A$ Receptor Modulators

Nova Syafni,[†,‖,‡] Maria Teresa Faleschini,[†,‡] Aleksandra Garifulina,[⁑] Ombeline Danton,[†] Mahabir P. Gupta,[⊤,+] Steffen Hering,[⁑] and Matthias Hamburger*,[†]

[†] Pharmaceutical Biology, Department of Pharmaceutical Sciences, University of Basel, Klingelbergstrasse 50, 4056 Basel, Switzerland

[‖] Faculty of Pharmacy and Sumatran Biota Laboratory, Andalas University, Kampus Limau Manis, Padang, West Sumatra, Indonesia

[⁑] Division of Pharmacology and Toxicology, Department of Pharmaceutical Sciences, University of Vienna, Pharmaziezentrum, Althanstrasse 14, 1090 Vienna, Austria

[⊤] Center for Pharmacognostic Research on Panamanian Flora, Faculty of Pharmacy, University of Panama City, Panama

Corresponding Author

Matthias Hamburger
Pharmaceutical Biology, Department of Pharmaceutical Sciences, University of Basel, Klingelbergstrasse 50, 4056 Basel, Switzerland
Email: matthias.hamburger@unibas.ch

Table of Contents

Figure S 1. Percentage of activation for 2 μM GABA (control), 200 μM GABA (100%), and diazepam (20 μM, in presence of 2 μM GABA), along with increasing concentrations of *Casearia corymbosa* extract (in presence of 2 μM GABA) (n=8, mean ± SEM). Final DMSO concentration in the assays was 0.1%. The * and *** above the bars indicate statistical significance with $p \leq 0.05$ and $p \leq 0.001$, respectively. 4

Table S 1. ^{1}H and ^{13}C NMR Spectroscopic Data for Compounds **2** and **3** ($CDCl_3$; 500.13 Hz for ^{1}H and 125.77 for ^{13}C NMR; δ in ppm). 5

Table S 2. ^{1}H and ^{13}C NMR Spectroscopic Data for Compounds **4** and **8** ($CDCl_3$; 500.13 Hz for 1H and 125.77 for ^{13}C NMR; δ in ppm)..6

Figure S 2. ^{1}H NMR spectrum of compound **1** in $CDCl_3$ (500 MHz)....................................7

Figure S 3. ^{1}H-^{1}H COSY spectrum of compound **1** in $CDCl_3$ (500 MHz).............................8

Figure S 4. HSQC spectrum of compound **1** in $CDCl_3$ (500 MHz). 9

Figure S 5. HMBC spectrum of compound **1** in $CDCl_3$ (500 MHz). 10

Figure S 6. ^{1}H-^{1}H NOESY spectrum compound **1** in $CDCl_3$ (500 MHz). 11

Figure S 7. ^{1}H NMR spectrum of compound **5** in $CDCl_3$ (500 MHz)..................................12

Figure S 8. ^{1}H-^{1}H COSY spectrum of compound **5** in $CDCl_3$ (500 MHz)...........................13

Figure S 9. HSQC spectrum of compound **5** in $CDCl_3$ (500 MHz). 14

Figure S 10. HMBC spectrum of compound **5** in $CDCl_3$ (500 MHz). 15

Figure S 11. ^{1}H-^{1}H NOESY spectrum of compound **5** in $CDCl_3$ (500 MHz). 16

Figure S 12. ^{1}H NMR spectrum of compound **6** in $CDCl_3$ (500 MHz)................................17

Figure S 13. ^{1}H-^{1}H COSY spectrum of compound **6** in $CDCl_3$ (500 MHz)..........................18

Figure S 14. HSQC spectrum of compound **6** in $CDCl_3$ (500 MHz). 19

Figure S 15. HMBC spectrum of compound **6** in $CDCl_3$ (500 MHz). 20

Figure S 16. ^{1}H-^{1}H NOESY spectrum of compound **6** in $CDCl_3$ (500 MHz). 21

Figure S 17. ^{1}H NMR spectrum of compound **7** in $CDCl_3$ (500 MHz)................................22

Figure S 18. ^{1}H-^{1}H COSY spectrum of compound **7** in $CDCl_3$ (500 MHz)..........................23

Figure S 20. HMBC spectrum of compound **7** in $CDCl_3$ (500 MHz). 25

Figure S 21. ^{1}H-^{1}H NOESY spectrum of compound **7** in $CDCl_3$ (500 MHz). 26

Figure S 22. ^{1}H NMR spectrum of compound **9** in $CDCl_3$ (500 MHz)................................27

Figure S 23. ^{1}H-^{1}H COSY spectrum of compound **9** in $CDCl_3$ (500 MHz)..........................28

Figure S 25. HMBC spectrum of compound **9** in CDCl3 (500 MHz)..................................30

Figure S 26. ^{1}H-^{1}H NOESY spectrum of compound **9** in $CDCl_3$ (500 MHz). 31

Figure S 27. Experimental ECD and UV spectra of compounds **2-4** and **6** in CH_3OH. 32

Figure S 28. Experimental ECD and UV spectra of compounds **7 - 9** in CH_3OH. 33

Figure S 29. Percentage of activation for 2 μM diazepam with increasing concentrations of flumazenil (in presence of 2 μM GABA), along with 2 μM GABA (control), 200 μM GABA (100%), and 2 μM diazepam (in presence of 2 μM GABA), (n = 4, mean ± SEM). Final DMSO concentration in the assay was 0.2%. The * and **** above the bars indicate statistical significance with $p \leq 0.05$, and $p \leq 0.0001$, respectively. 34

Figure S 30. Percentage of activation for 0.5 μM allopregnanolone with increasing concentrations of PREGS (in presence of 2 μM GABA), along with 2 μM GABA (control), 200 μM GABA (100%), and 0.5 μM allopregnanolone (in presence of 2 μM GABA) (n = 4, mean ± SEM). Final DMSO concentration in the assay was 0.2%. The **** above the bars indicate statistical significance with $p \leq 0.0001$...34

Figure S 31. Percentage of activation for increasing concentrations of etazolate (in presence of 2 μM GABA), along with 2 μM GABA (control), 200 μM GABA (100%), and 20 μM diazepam (in presence of 2 μM GABA), (n = 4, mean ± SEM). Final DMSO concentration in the assay was 0.2%. The ** and **** above the bars indicate statistical significance with $p \leq 0.01$, and $p \leq 0.0001$, respectively...35

Figure S 32. Percentage of activation for increasing concentrations of ethanol (in presence of 2 μM GABA), along with 2 μM GABA (control), 200 μM GABA (100%), and 20 μM diazepam (in presence of 2 μM GABA), (n = 4, mean ± SEM)...36

Figure S 33. Percentage of activation by (**A**) **8** (5 μM) and increasing concentrations of flumazenil (in presence of 2 μM GABA), and (**B**) diazepam (2 μM, in the presence of 2 μM GABA) and increasing concentrations of **8** (in presence of 2 μM GABA), together with 2 μM GABA (control), 200 μM GABA (100%), and 2 μM diazepam (in presence of 2 μM GABA; positive control), (n = 4, mean ± SEM). Final DMSO concentration in the assays was 0.2%. The **** above the bars indicate statistical significance with $p \leq 0.0001$ 36

Figure S 34. Percentage of activation by etazolate (0.78 μM) and increasing concentrations of 8 (in presence of 2 μM GABA), along with 2 μM GABA (control), 200 μM GABA (100%), and 0.78 μM etazolate (in presence of 2 μM GABA; positive control), (n = 4, mean ± SEM). Final DMSO concentration in the assay was 0.2%. The *, ** and *** above the bars indicate statistical significance with $p \leq 0.05$, $p \leq 0.01$ and $p \leq 0.001$, respectively. 37

Figure S 35 Figure 8. Percentage of activation (**A**) with compound **8** (10 μM) and increasing concentrations of PREGS (in presence of 2 μM GABA), and (**B**) with allopregnanolone (0.25 μM) and increasing concentrations of **8** (in presence of 2 μM GABA), along with 2 μM GABA (control), 200 μM GABA (100%), and 0.25 μM allopregnanolone (in presence of 2 μM GABA; positive control) (n=4, mean ± SEM). Final DMSO concentration in the assay was 0.2%. The **** above the bars indicate statistical significance with $p \leq 0.0001$. 38

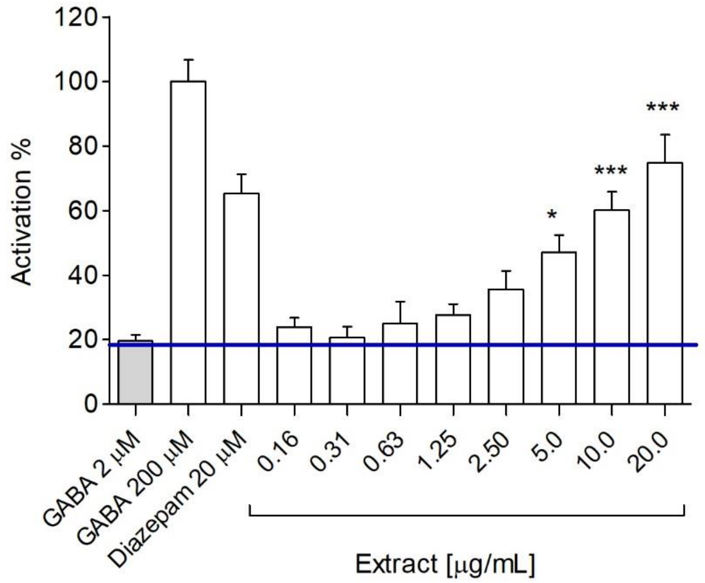

Figure S 1. Percentage of activation for 2 µM GABA (control), 200 µM GABA (100%), and diazepam (20 µM, in presence of 2 µM GABA), along with increasing concentrations of *Casearia corymbosa* extract (in presence of 2 µM GABA) (n = 8, mean ± SEM). Final DMSO concentration in the assays was 0.1%. The * and *** above the bars indicate statistical significance with $p \leq 0.05$ and $p \leq 0.001$, respectively.

Table S 1. ^1H and ^{13}C NMR Spectroscopic Data for Compounds **2** and **3** ($CDCl_3$; 500.13 Hz for ^1H and 125.77 for ^{13}C NMR; δ in ppm).

position	**2**		**3**	
	$\delta_C{}^a$, type	δ_H (J in Hz)	$\delta_C{}^a$, type	δ_H (J in Hz)
1α<br>1β	26.3, CH_2	2.18^b<br>1.66^b	26.2, CH_2	2.15^b<br>1.68^b
2	71.0, CH	5.58, dddd (9.6, 6.7, 2.4, 1.5)	70.8, CH	5.58, dddd (9.2, 7.0, 2.3, 1.8)
3	123.6, CH	5.98, br s	123.8, CH	5.89, br s
4	145.7, C	-	144.8, C	-
5	53.5, C	-	53.5, C	-
6	74.1, CH	3.96, ddd (11.9, 8.5, 3.7)	73.9, CH	3.97, ddd (11.6, 7.6, 4.3)
7	37.8, CH_2	1.64^b<br>1.76, ddd (13.4, 3.7, 3.7)	37.6, CH_2	1.63^b<br>1.73^b
8	36.7, CH	1.84, m	36.7, CH	1.84, m
9	38.5, C	-	38.4, C	-
10	41.5, CH	2.37, dd (14.0, 2.8)	41.6, CH	2.36, dd (14.0, 2.8)
11	30.1, CH_2	1.70, m<br>2.22^b	30.0, CH_2	1.66^b<br>2.20^b, dd (16.8, 7.9)
12	128.9, CH	5.38, br dd (7.6, 3.1)	128.8, CH	5.36, br dd (6.7, 2.8)
13	135.6, C	-	135.8, C	-
14	141.2, CH	6.30, dd (17.2, 10.8)	141.2, CH	6.29, dd (17.1, 10.7)
15	110.7, CH_2	4.93, d (10.7)<br>5.08, d (17.4)	110.8, CH_2	4.91, d (10.7)<br>5.07, d (17.4)
16	11.8, CH_3	1.66^b	11.8, CH_3	1.64^b
17	15.5, CH_3	0.93, d (6.7)	15.5, CH_3	0.91, d (6.7)
18	104.2, CH	5.46, dd (1.5, 1.5)	95.2, CH	6.68, dd (1.8, 1.4)
19	96.4, CH	6.44, s	96.8, CH	6.45, s
20	24.9, CH_3	0.84, s	24.8, CH_3	0.83, s
1’	170.5, C	-	170.7, C	-
2’	21.0, CH_3	2.06, s	21.0, CH_3	2.05^b
6-OR1	-	2.11, d (9.2)	-	2.15^b
2	-	-		
18-OR1	55.8, CH_3	3.42, s	169.9, C	-
2	-	-	21.0,- CH_3	2.05^b
19-OR1	169.7, C	-	169.2, C	-
2	21.5, CH_3	1.93, s	21.4, CH_3	1.91, s

^{a} ^{13}C NMR data extracted from HSQC and HMBC spectra, ^{b}Overlapping signals.

Table S 2. ^{1}H and ^{13}C NMR Spectroscopic Data for Compounds **4** and **8** ($CDCl_3$; 500.13 Hz for 1H and 125.77 for ^{13}C NMR; δ in ppm).

position	4		8	
	δ_C, type	δ_H (J in Hz)	δ_C, type	δ_H (J in Hz)
1α<br>1β	26.7, CH_2	1.94[b]<br>1.87[b]	26.4, CH_2	2.16[b]<br>1.69[b]
2	66.7, CH	5.44, br dd (3.8, 3.8)	70.9, CH	5.60, dddd (9.2, 7.0, 2.4, 1.8)
3	121.6, CH	5.96, br d (3.7)	123.4, CH	5.85, br s
4	145.5, C	-	145.3, C	-
5	53.5, C	-	52.9, C	-
6	72.6, CH	3.78, ddd (11.0, 7.0, 4.0)	82.8, CH	3.50, dd (12.1, 3.8)
7	37.3, CH_2	1.61[b]<br>1.67[b]	31.6, CH_2	1.44, ddd (12.8, 12.8, 12.8)<br>1.88, ddd (13.7, 3.7, 3.5)
8	36.7, CH	1.74, m	36.2, CH	1.77, m
9	37.6, C	-	38.5, C	-
10	36.9, CH	2.33, dd (13.1, 3.4)	41.5, CH	2.39, dd (13.9, 2.6)
11	30.3, CH_2	2.18, dd (16.9, 8.1)<br>1.66[b]	29.9, CH_2	1.65[b]<br>2.20, dd (17.7, 8.2)
12	129.0, CH	5.34, br dd (7.3, 3.1)	129.0, CH	5.36, br dd (7.6, 2.4)
13	135.7, C	-	135.7, C	-
14	141.2, CH	6.21, dd (17.4, 10.7)	141.2, CH	6.29, dd (17.1, 10.7)
15	110.7, CH_2	5.02, d (17.4)<br>4.86, d (10.7)	110.9, CH_2	4.93, d (11.0)<br>5.07, d (17.1)
16	12.0, CH_3	1.61[b]	11.9, CH_3	1.64[b]
17	15.5, CH_3	0.88, d (6.7)	15.7, CH_3	0.94, d (7.0)
18	95.6, CH	6.65, br s	95.7, CH	6.61, dd (1.5, 1.5)
19	97.0, CH	6.47, s	97.1, CH	6.42, s
20	25.0, CH_3	0.76, s	25.1, CH_3	0.82, s
1’	166.6, C	-	170.8, C	-
2’	136.6, C	-	21.2, CH_3	2.09[b]
3’	125.5, CH_2	5.57, br s<br>6.10, br s	-	-
4’	18.3, CH_3	1.94[b]	-	-
6-OR	-	2.88, d (7.6)	57.5, CH_3	3.33, s
18-OR1	170.7, C	-	170.3, C	-
2	21.2, CH_3	2.03, s	21.2, CH_3	2.08[b]
19-OR1	169.3, C	-	169.5, C	-
2	21.6, CH_3	1.86[b]	21.7, CH_3	1.92, s

[a] ^{13}C NMR data extracted from HSQC and HMBC spectrum, [b] Overlapping signals.

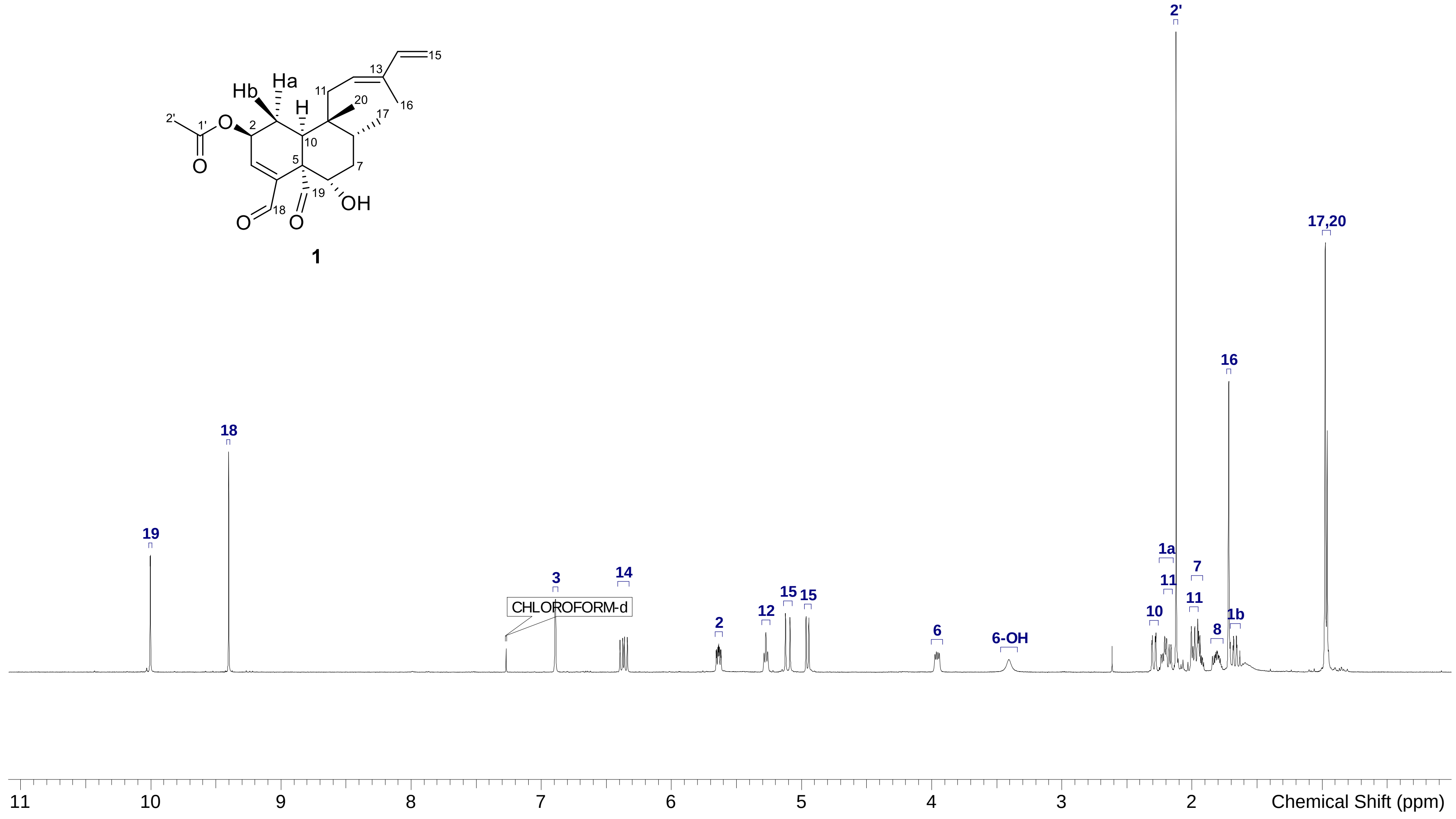

Figure S 2. ^{1}H NMR spectrum of compound **1** in $CDCl_3$ (500 MHz).

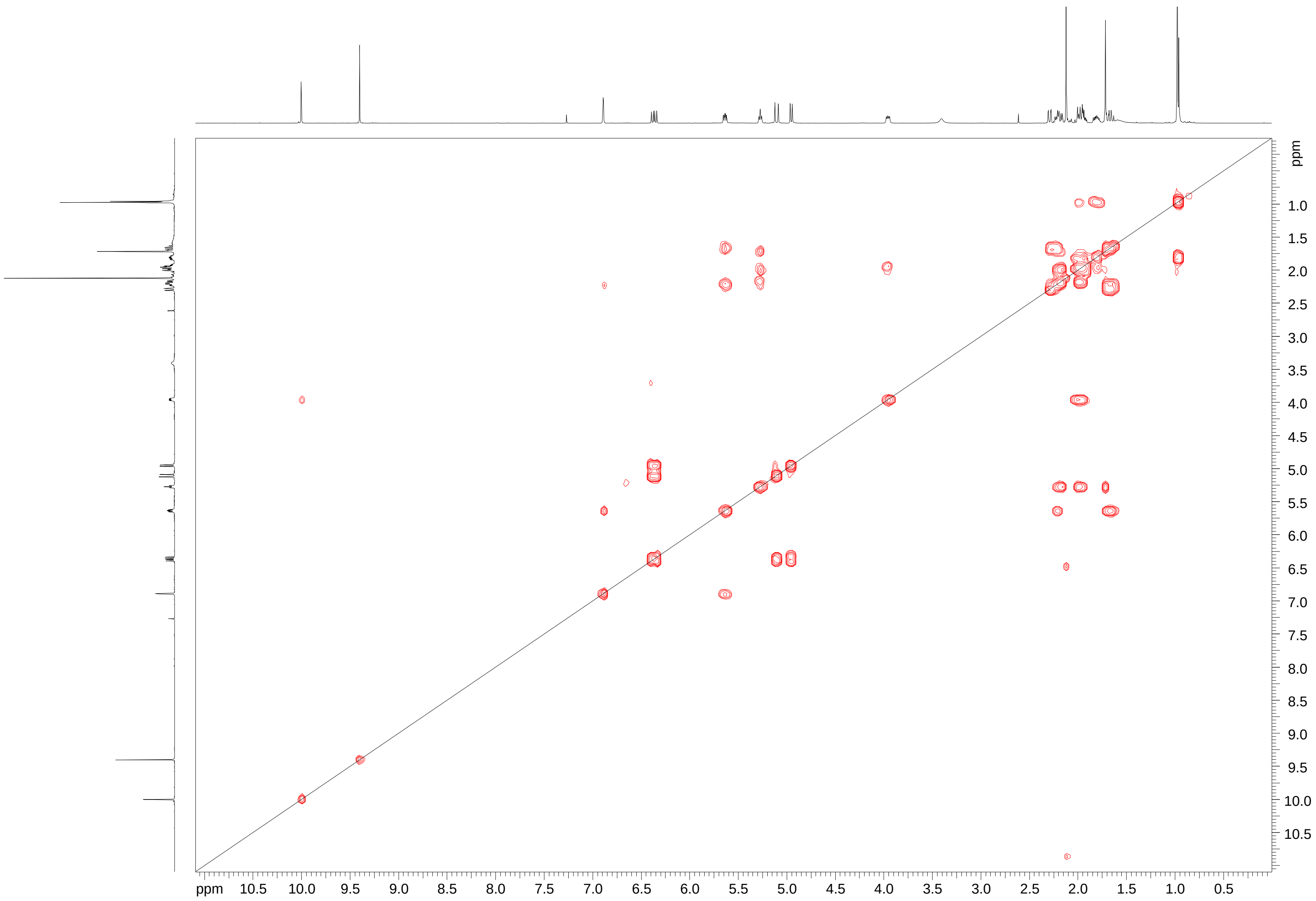

Figure S 3. ^{1}H-^{1}H COSY spectrum of compound **1** in $CDCl_3$ (500 MHz).

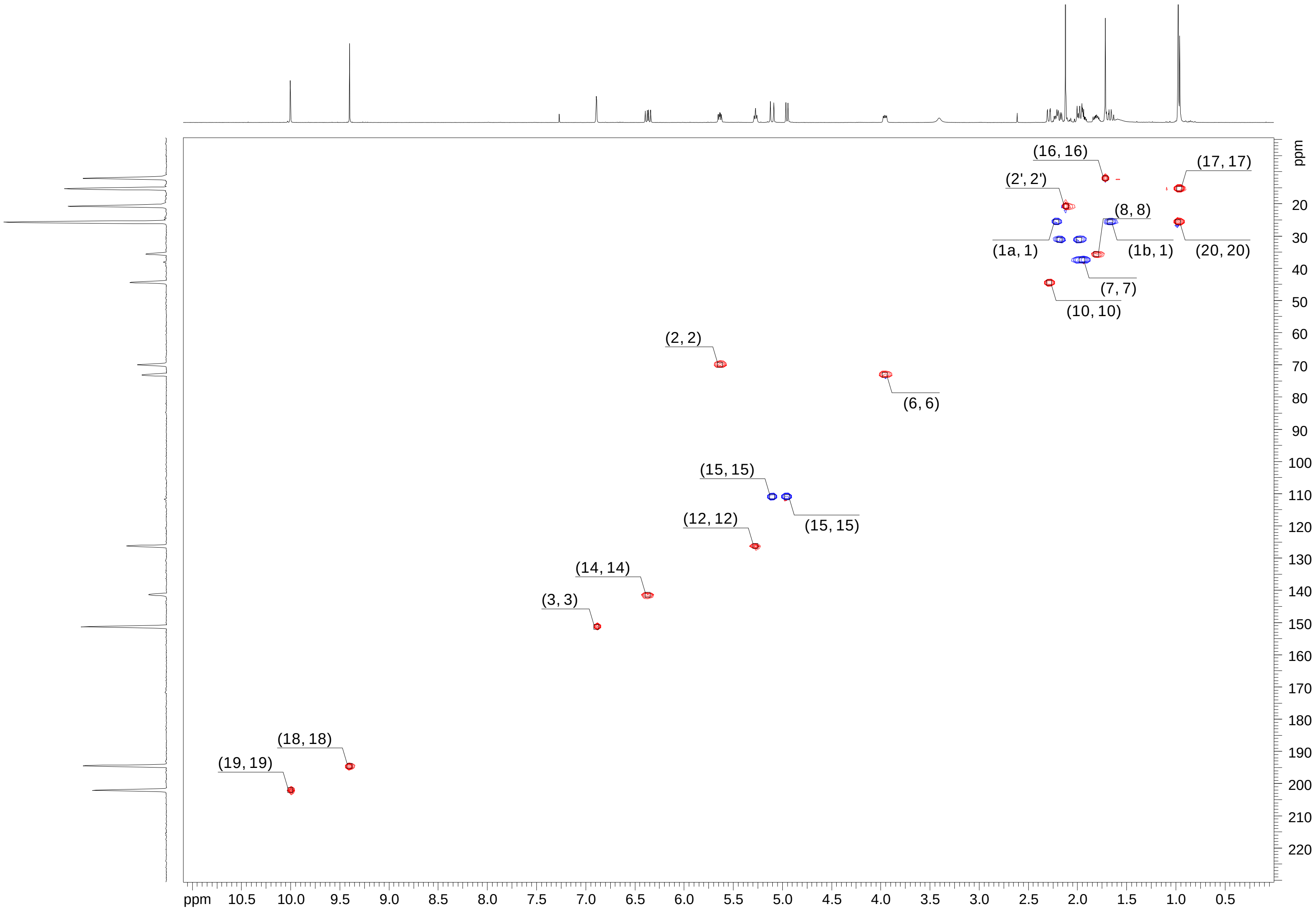

Figure S 4. HSQC spectrum of compound **1** in $CDCl_3$ (500 MHz).

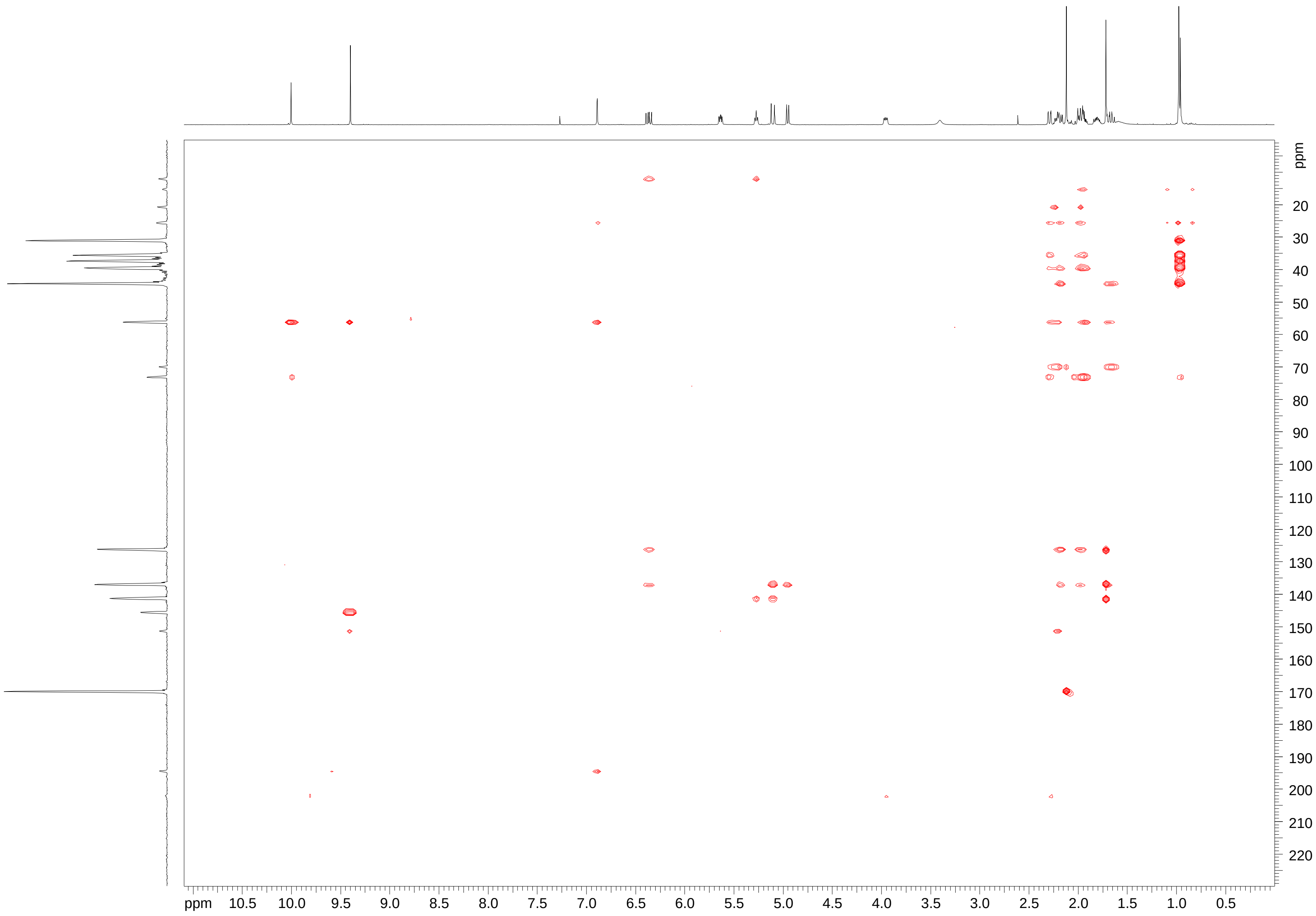

Figure S 5. HMBC spectrum of compound **1** in $CDCl_3$ (500 MHz).

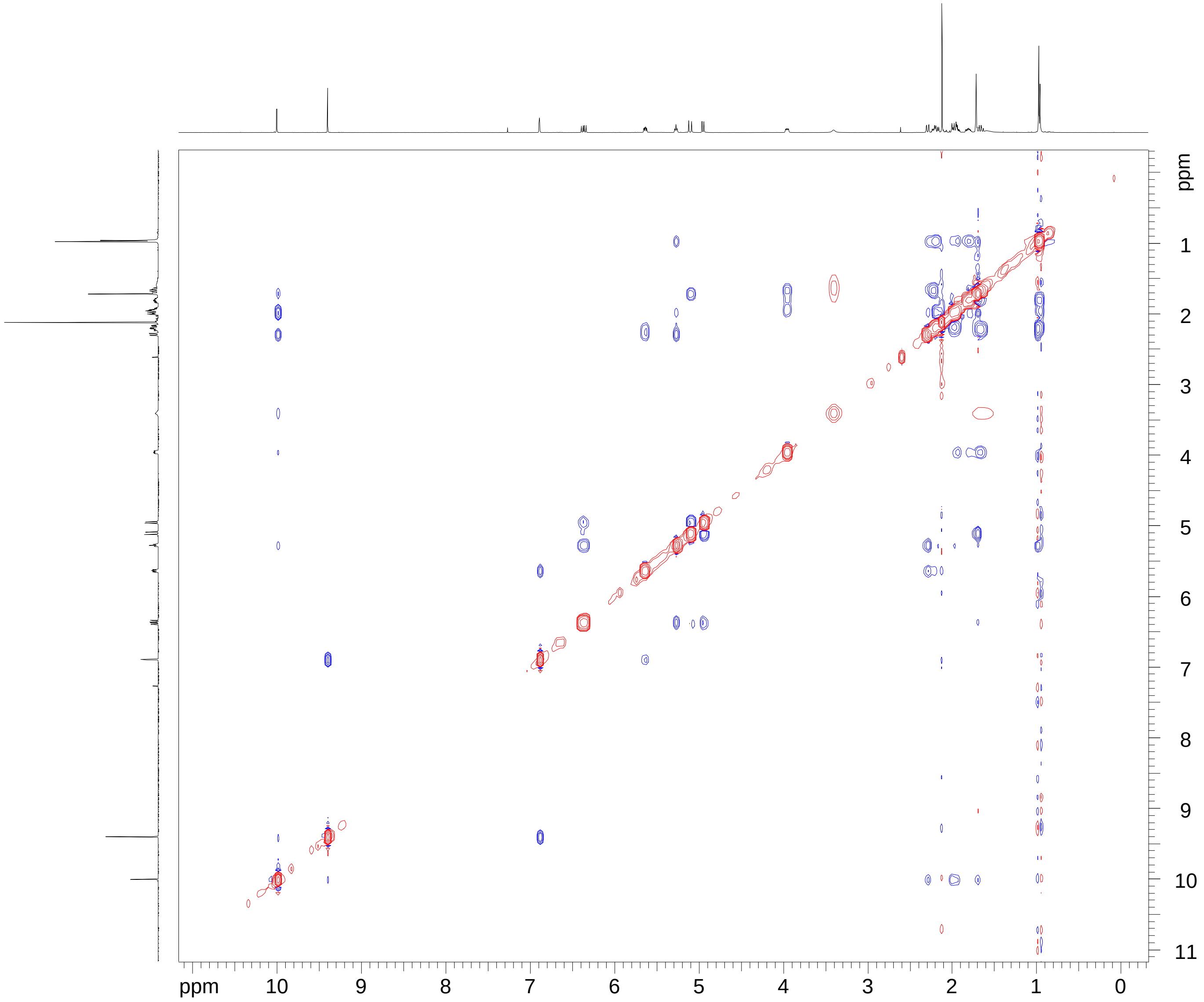

Figure S 6. ^{1}H-^{1}H NOESY spectrum compound **1** in $CDCl_3$ (500 MHz).

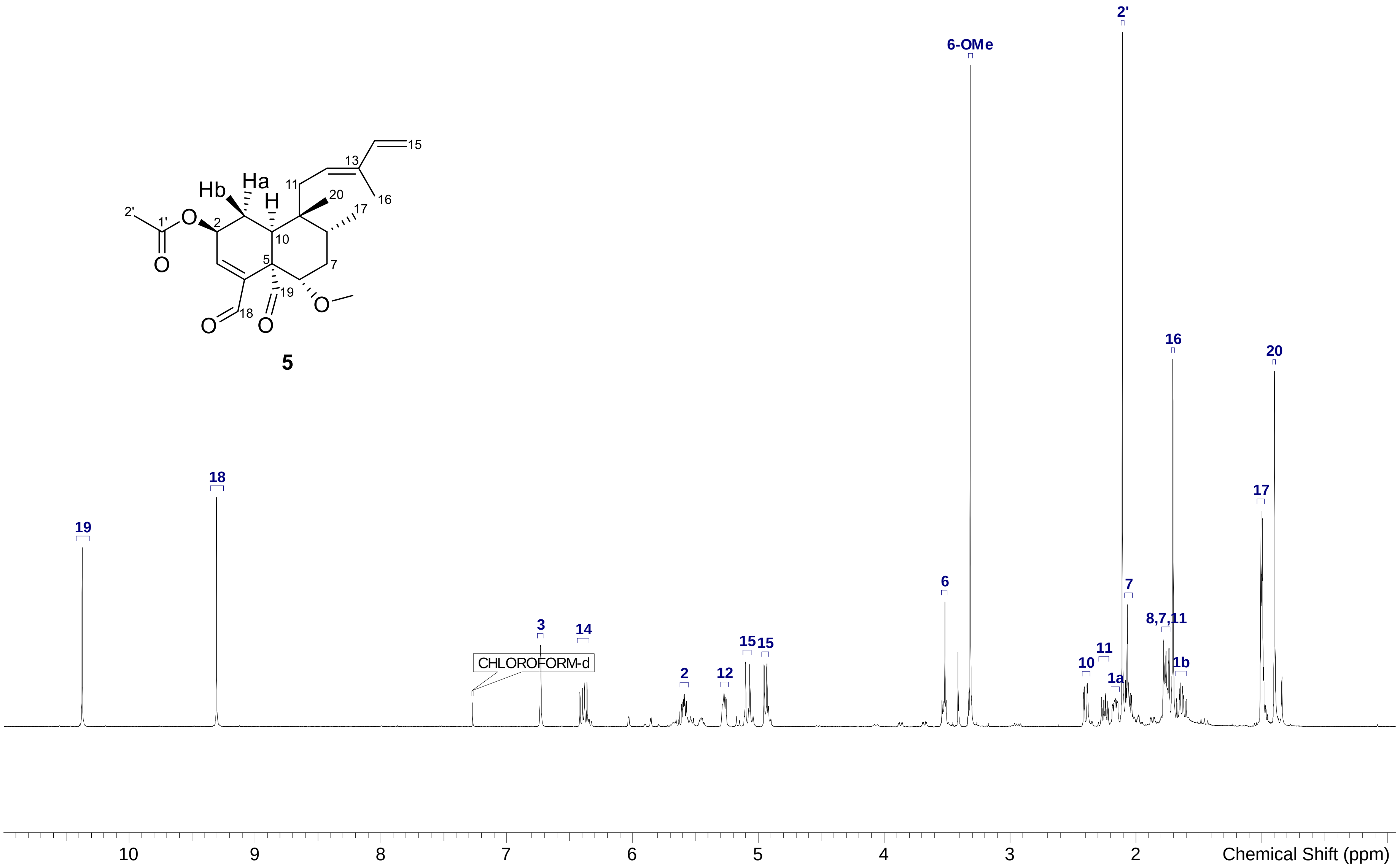

Figure S 7. ^{1}H NMR spectrum of compound **5** in $CDCl_3$ (500 MHz).

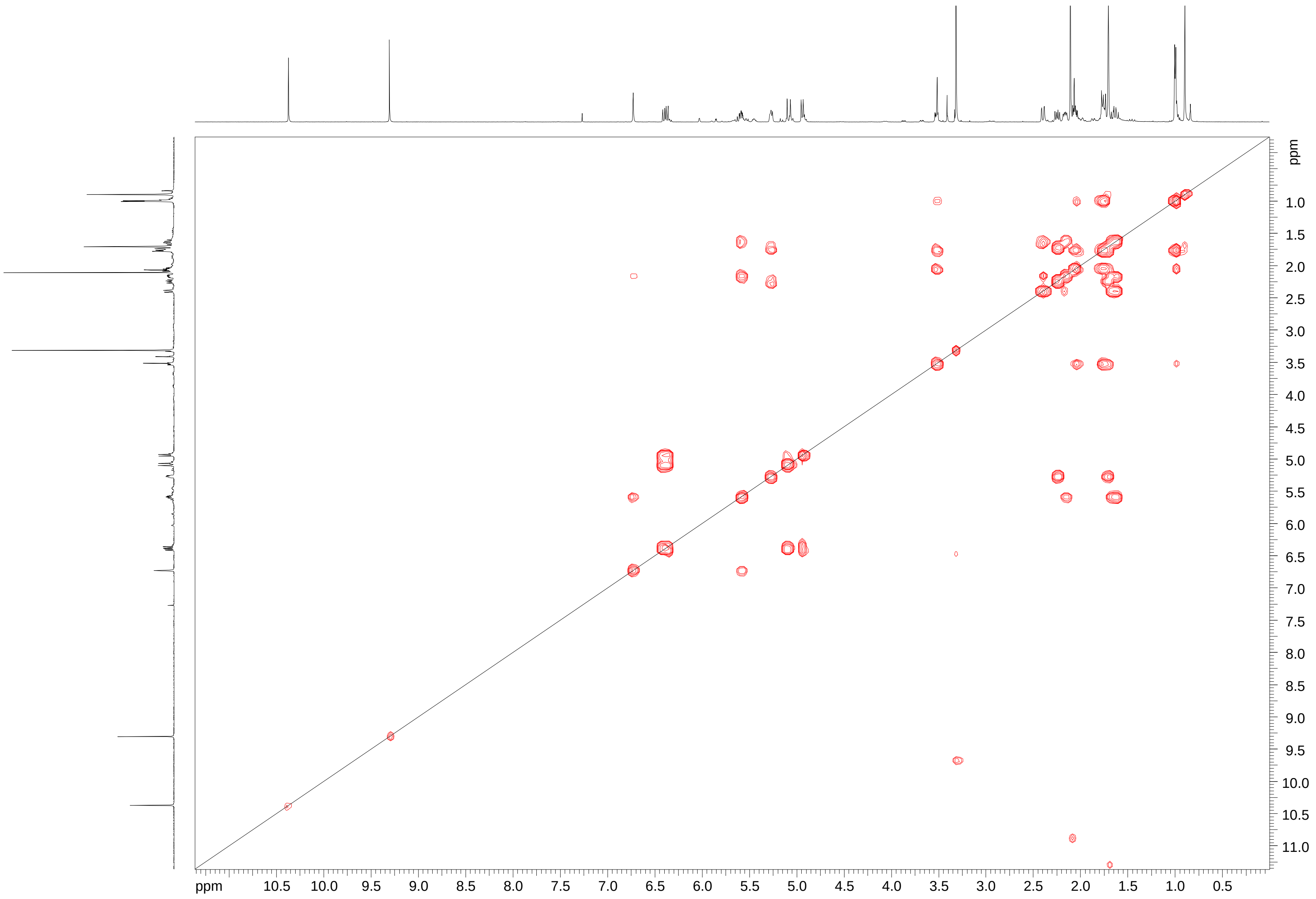

Figure S 87. ^{1}H-^{1}H COSY spectrum of compound **5** in $CDCl_3$ (500 MHz).

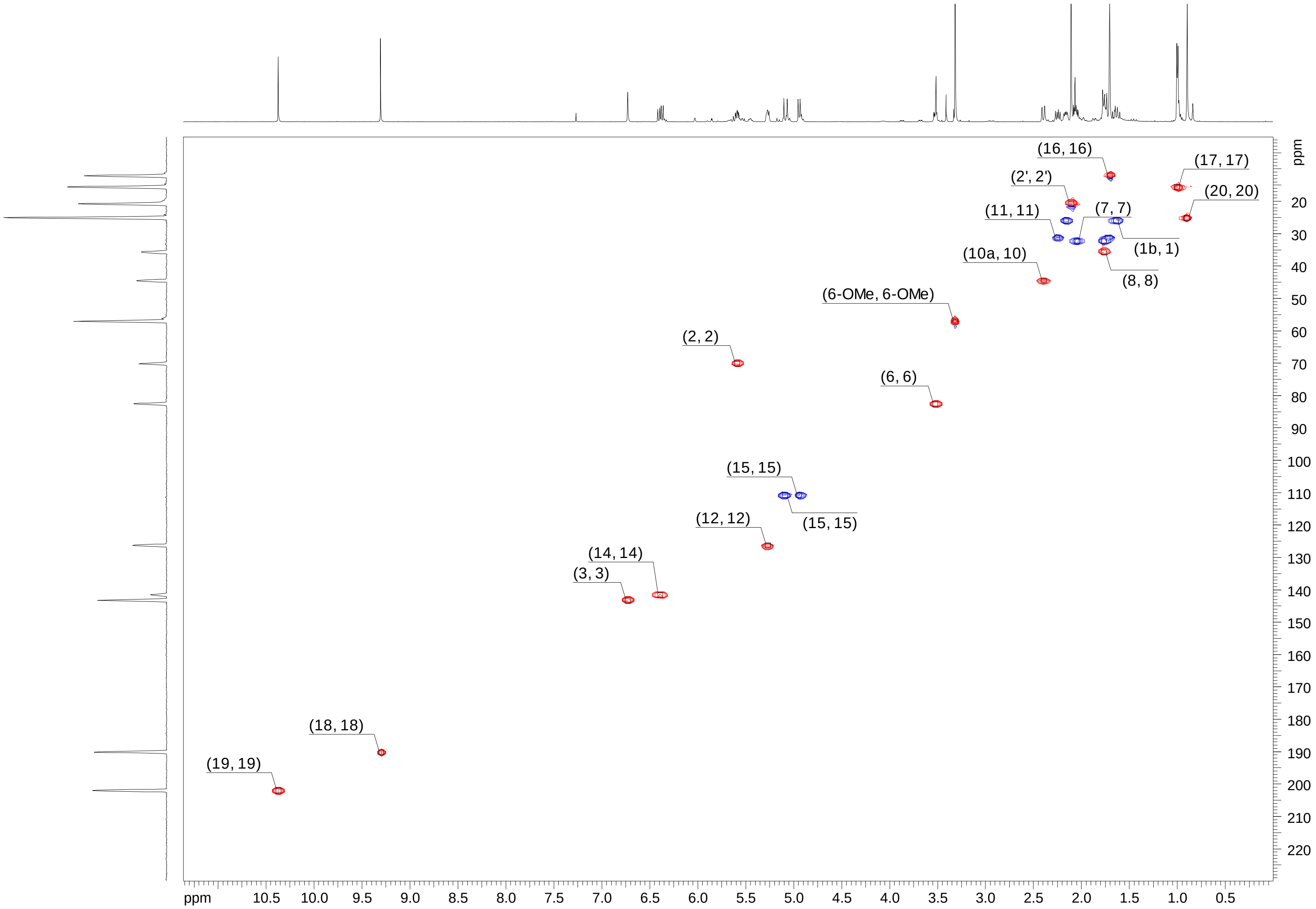

Figure S 8. HSQC spectrum of compound **5** in $CDCl_3$ (500 MHz).

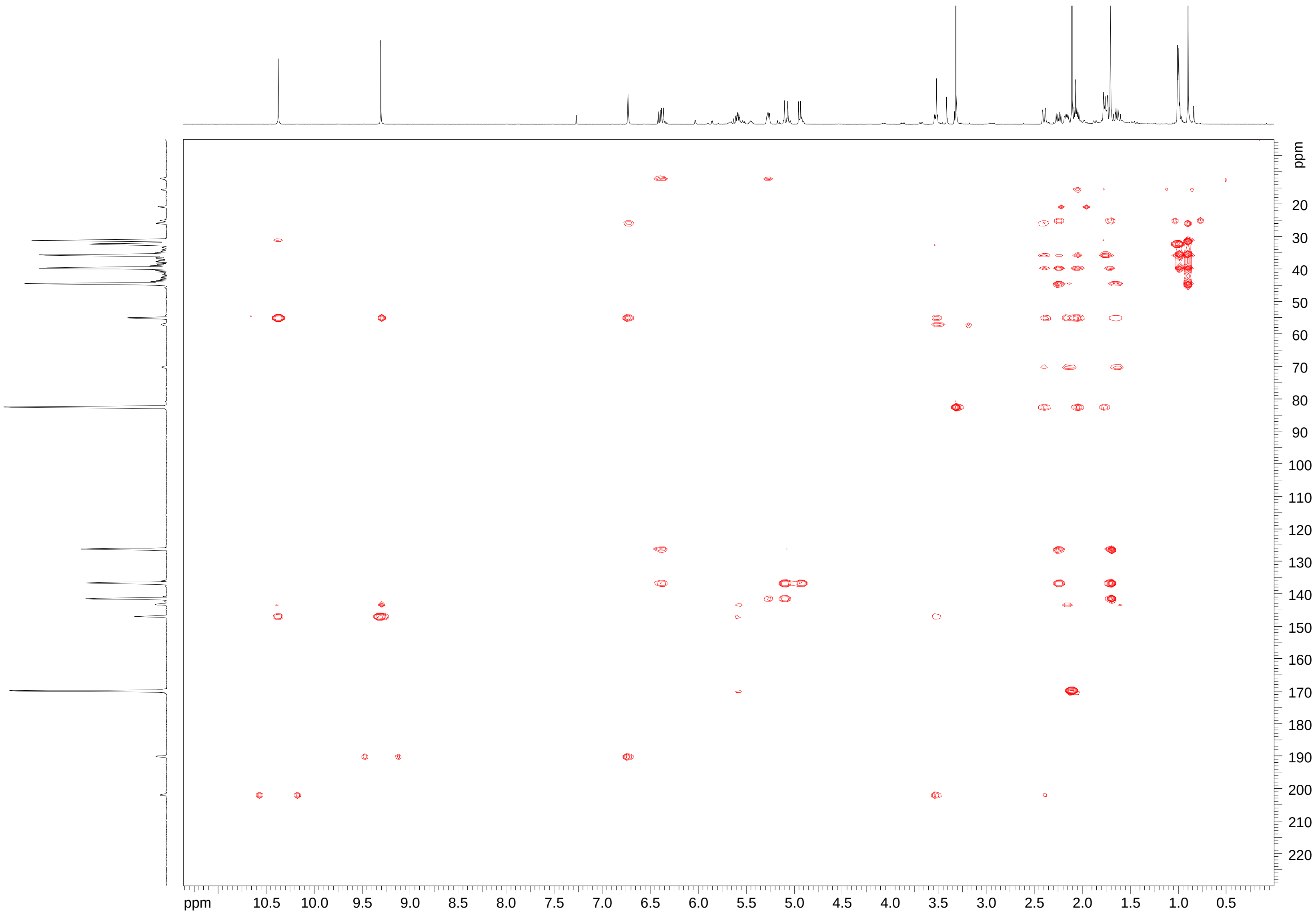

Figure S 90. HMBC spectrum of compound **5** in $CDCl_3$ (500 MHz).

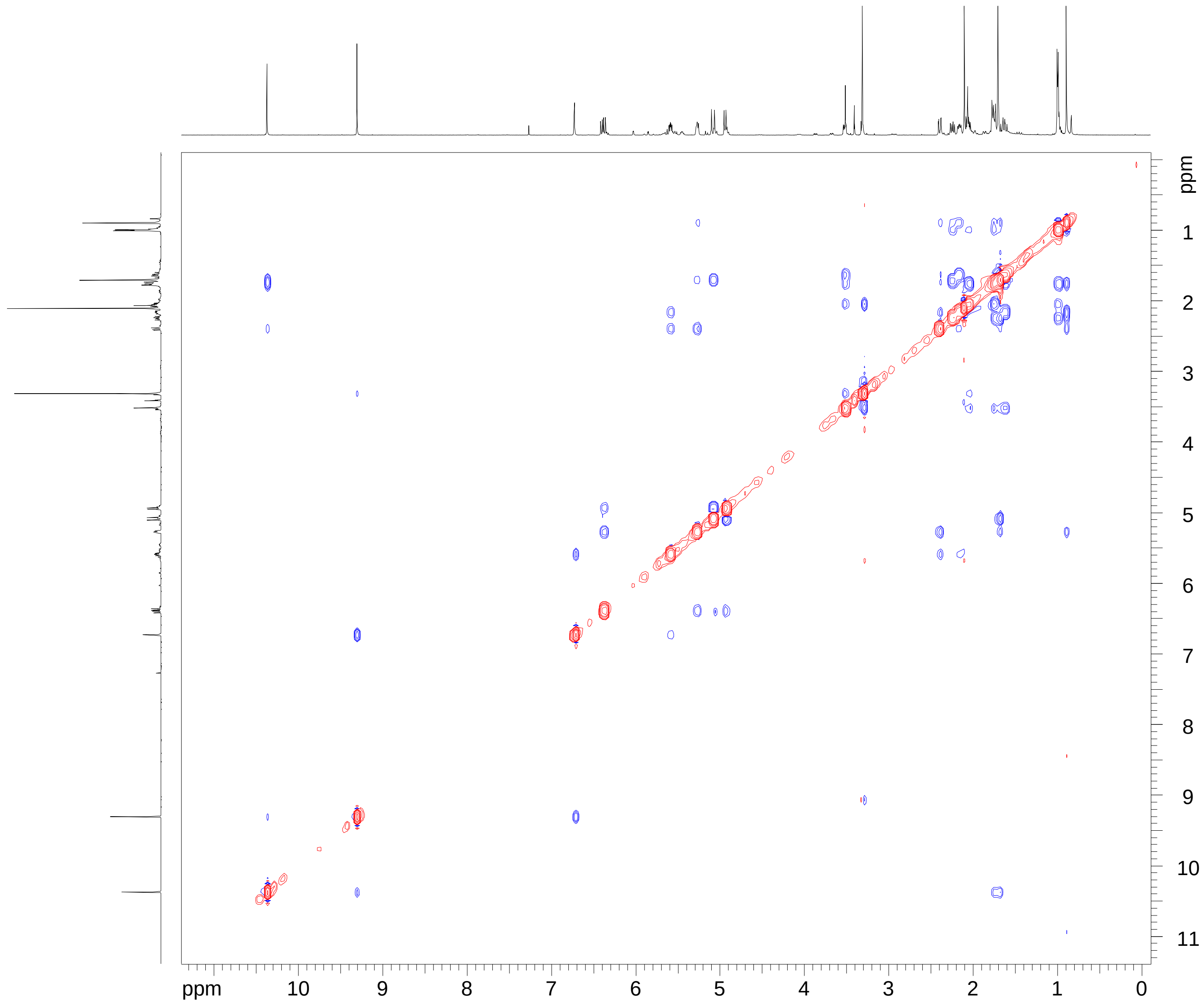

Figure S 101. ^{1}H-^{1}H NOESY spectrum of compound **5** in $CDCl_3$ (500 MHz).

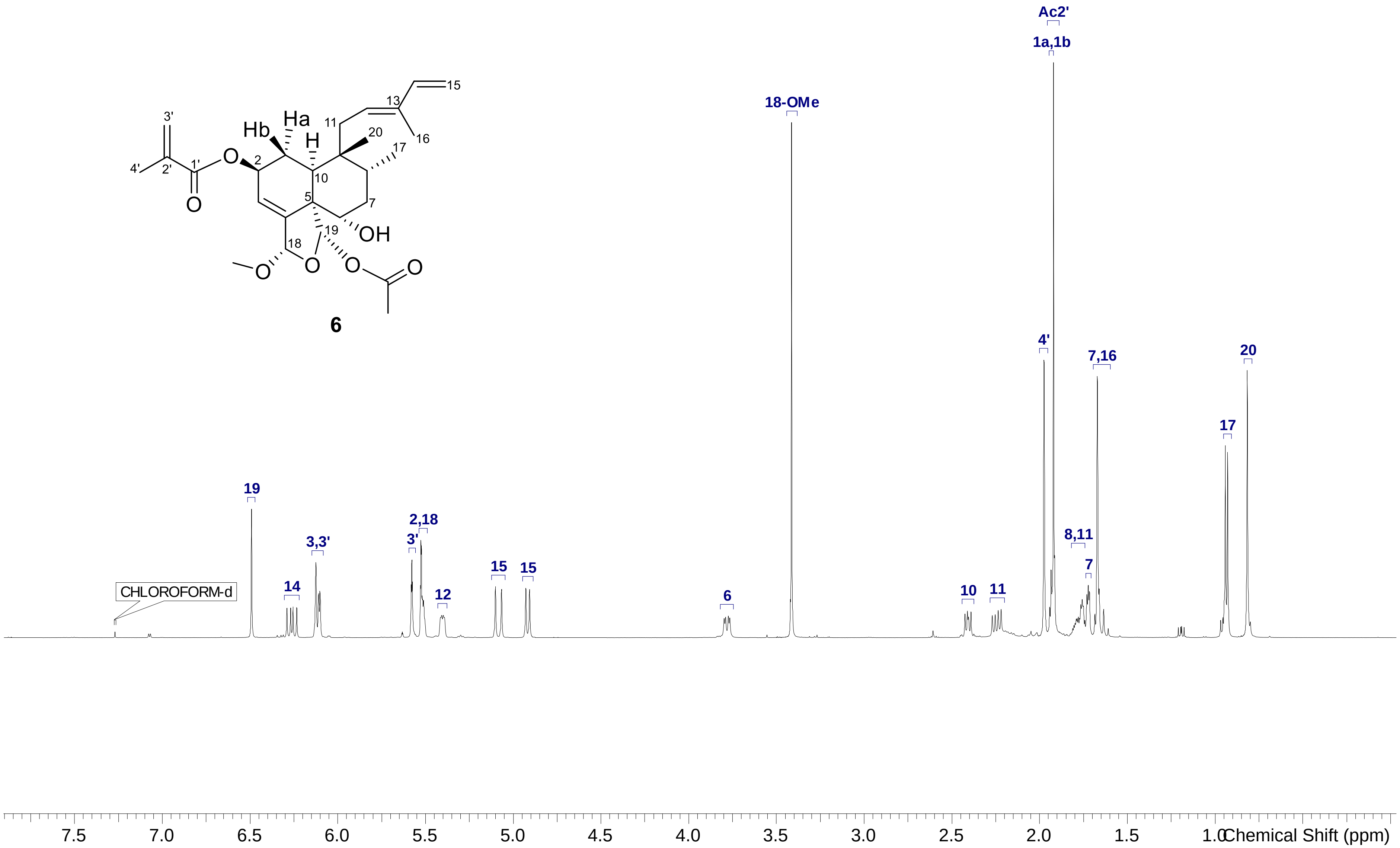

Figure S 11. ^{1}H NMR spectrum of compound **6** in $CDCl_3$ (500 MHz).

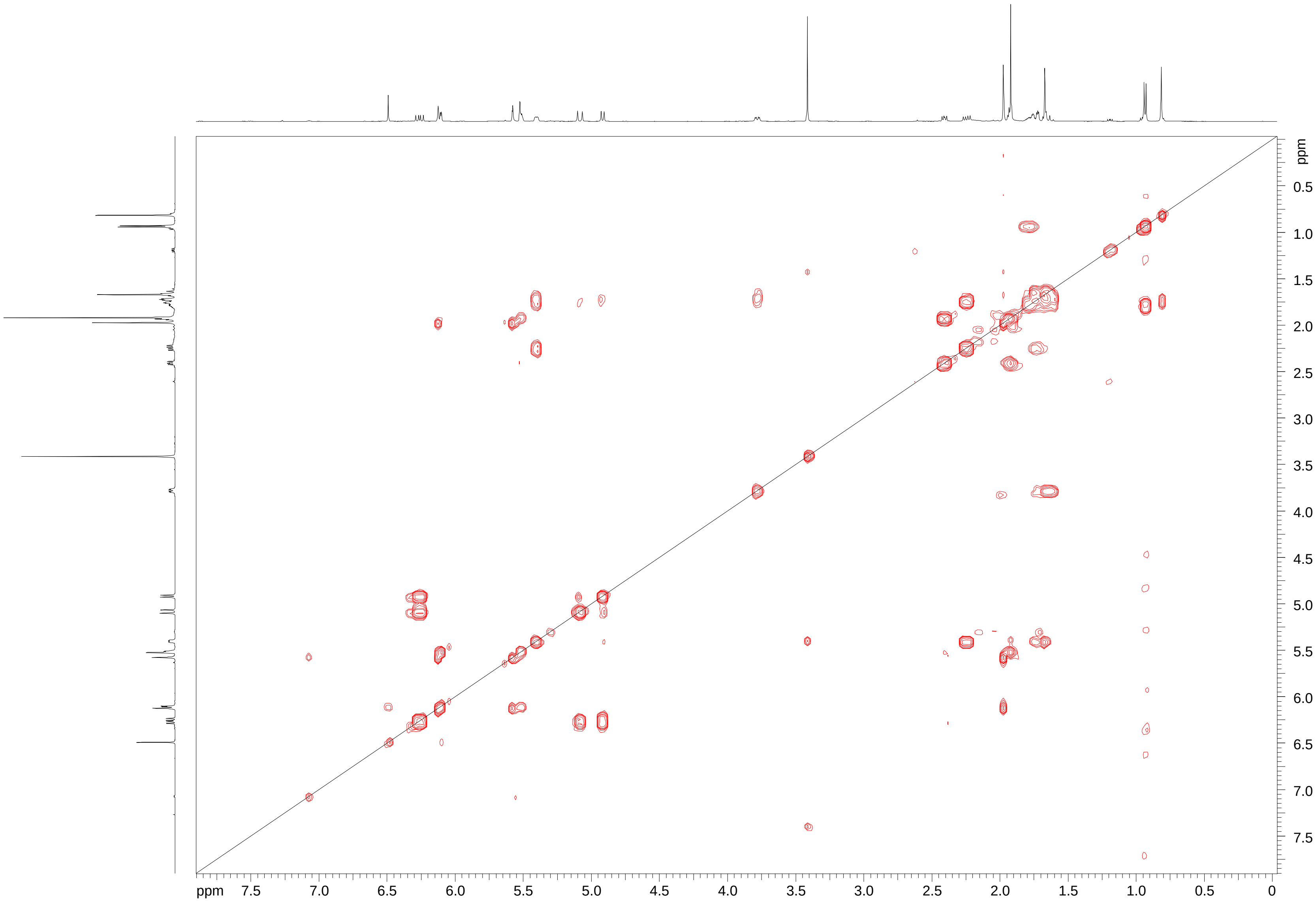

Figure S 123. ^{1}H-^{1}H COSY spectrum of compound **6** in $CDCl_3$ (500 MHz).

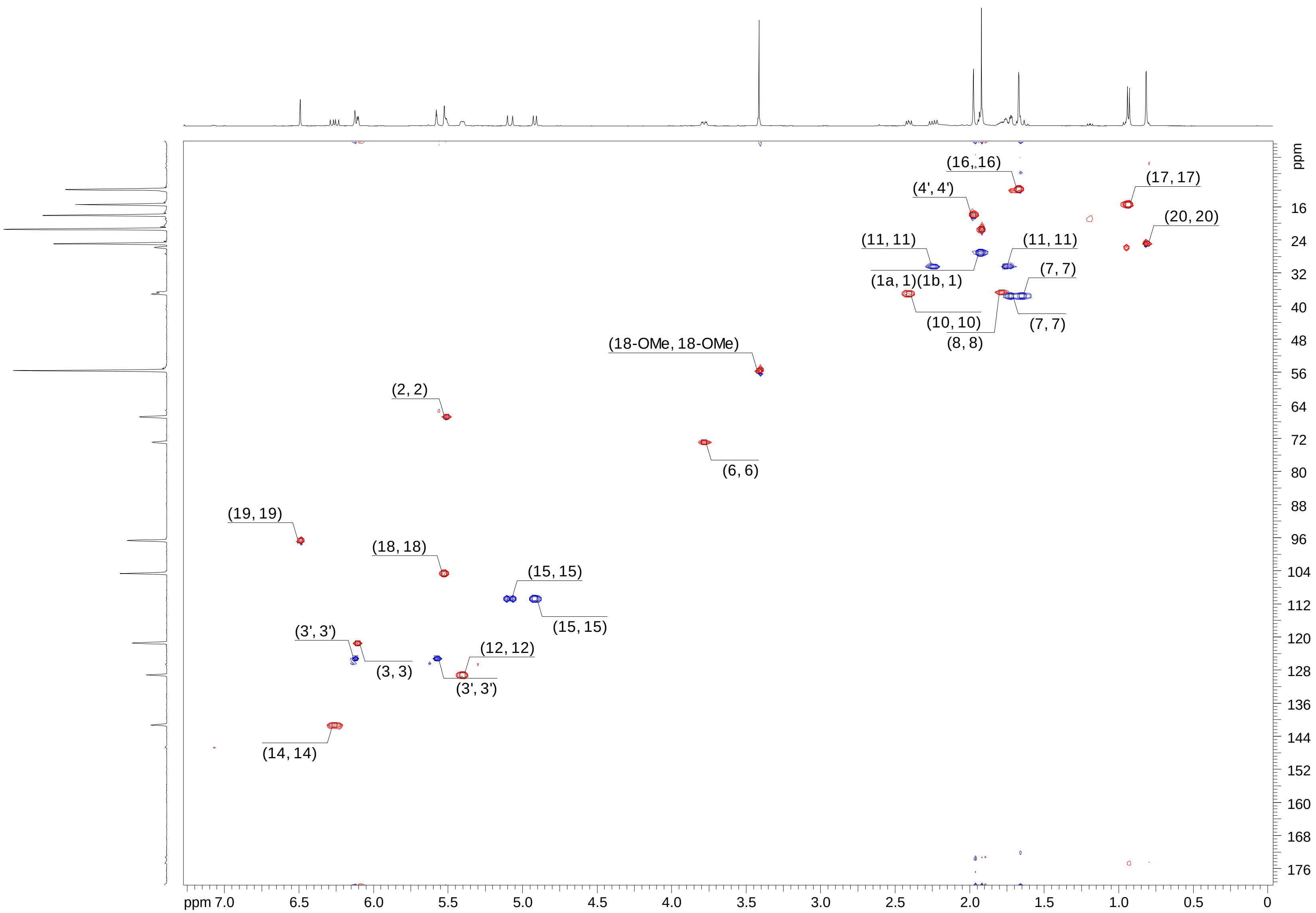

Figure S 134. HSQC spectrum of compound **6** in CDCl3 (500 MHz).

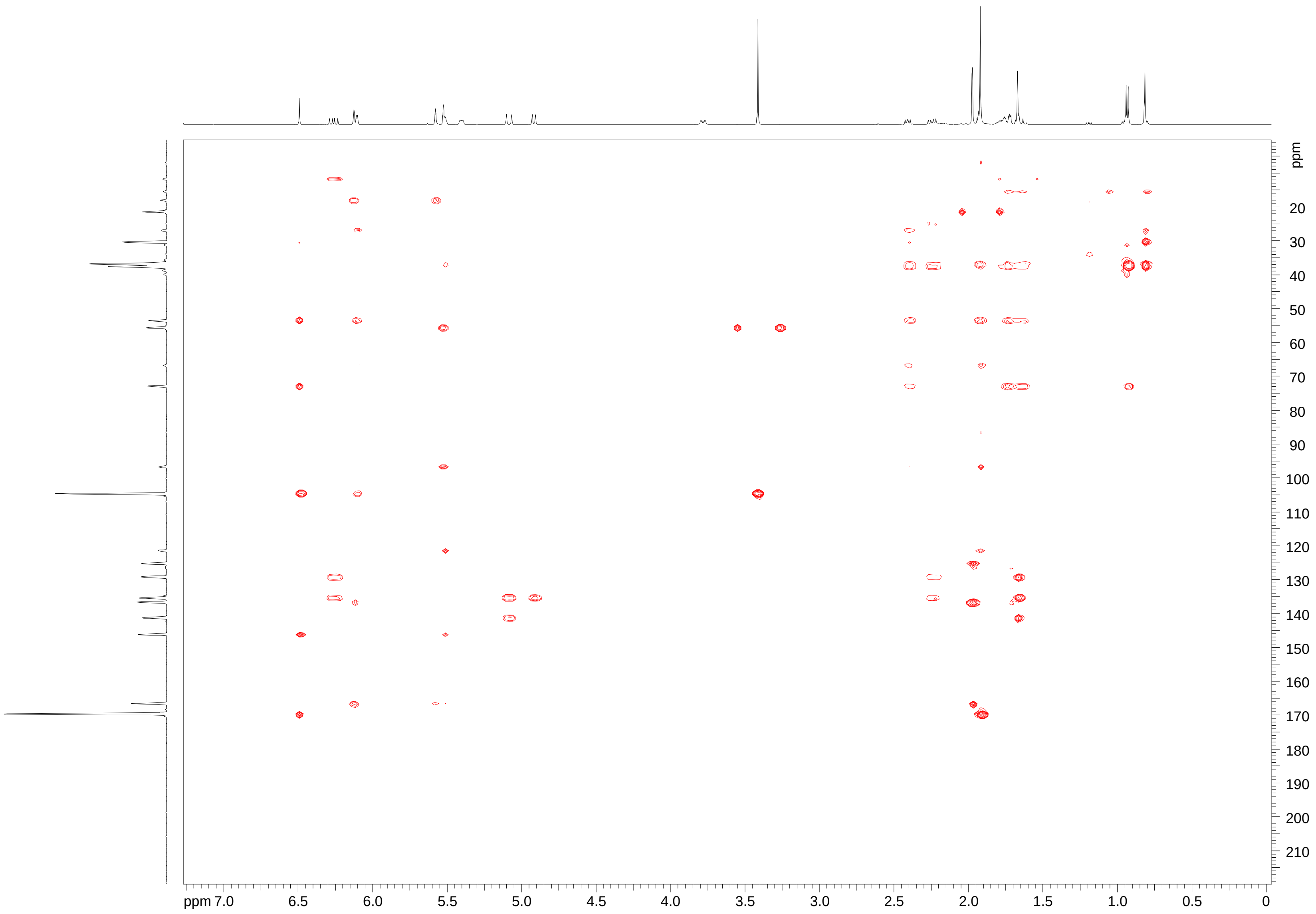

Figure S 14. HMBC spectrum of compound **6** in $CDCl_3$ (500 MHz).

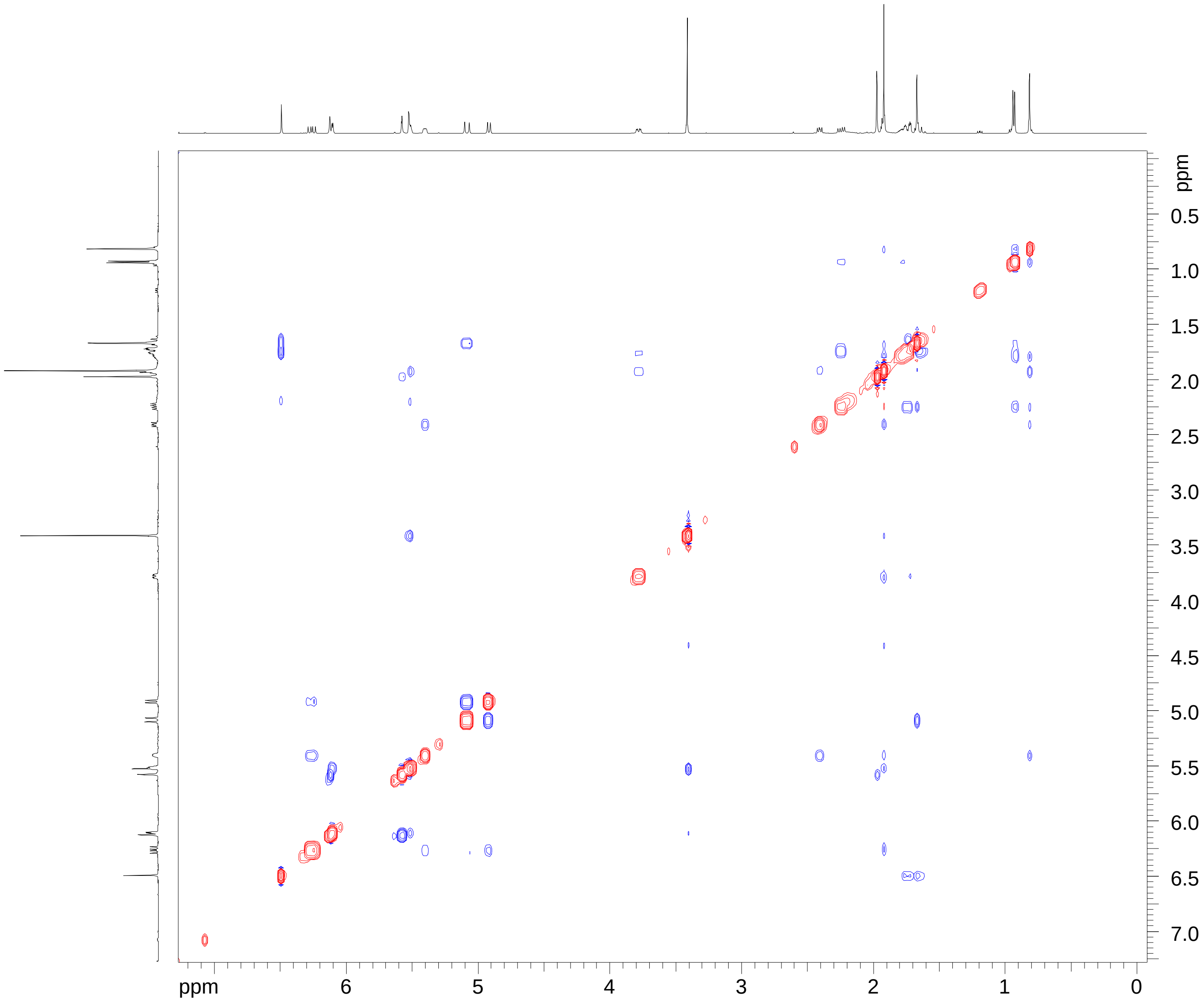

Figure S 16. ^{1}H-^{1}H NOESY spectrum of compound **6** in $CDCl_3$ (500 MHz).

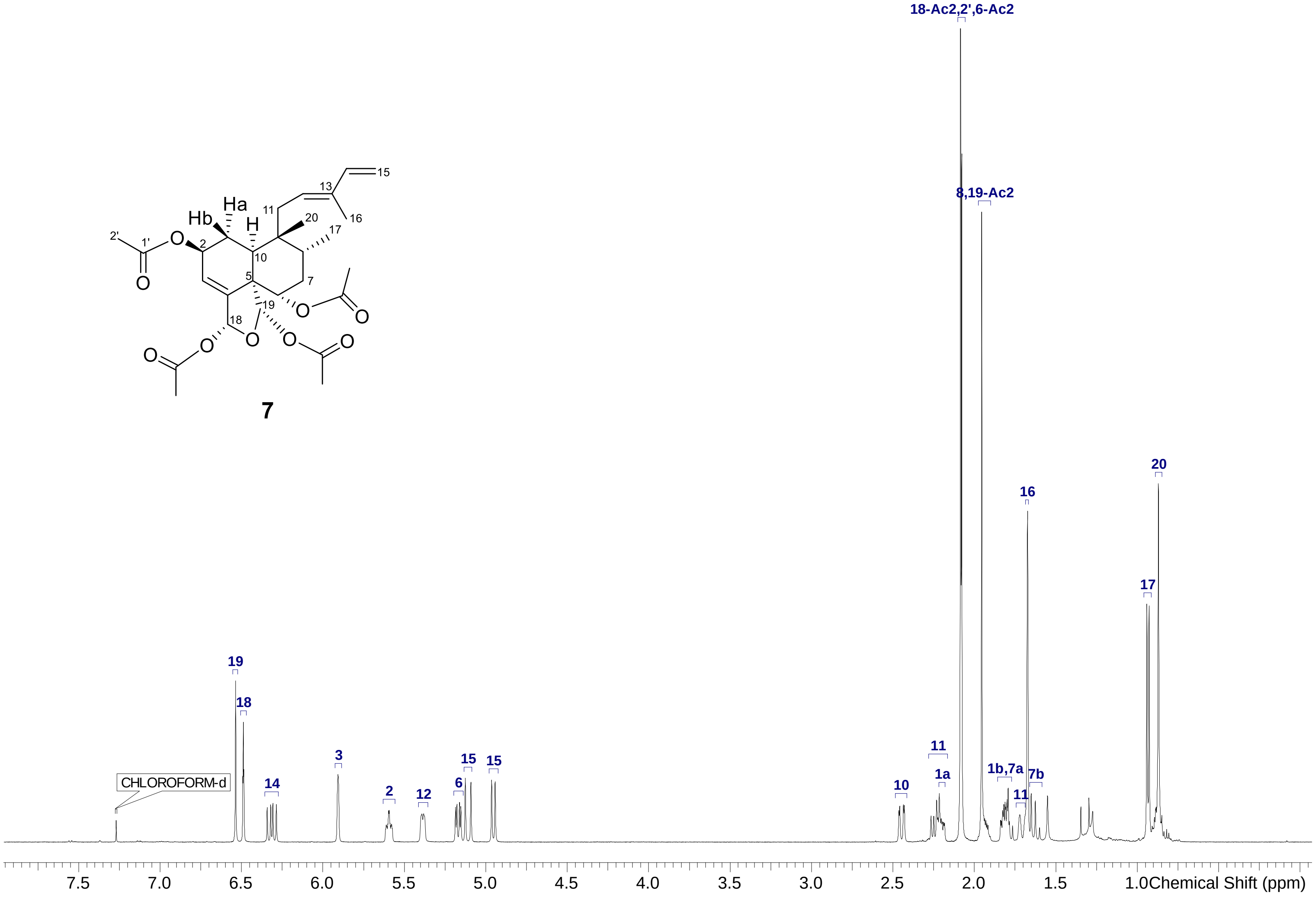

Figure S 15. ^{1}H NMR spectrum of compound **7** in $CDCl_3$ (500 MHz).

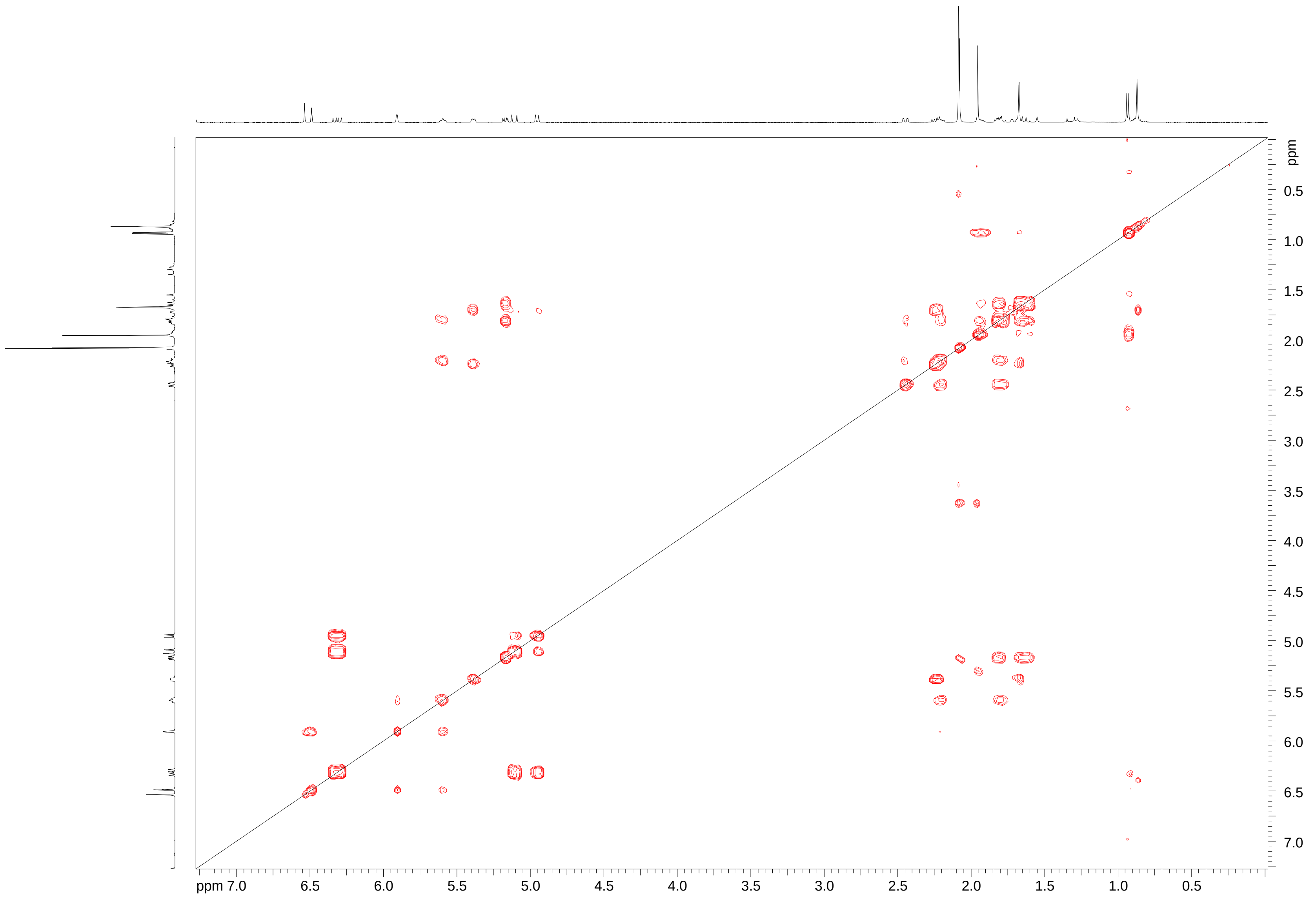

Figure S 18. ^{1}H-^{1}H COSY spectrum of compound **7** in $CDCl_3$ (500 MHz).

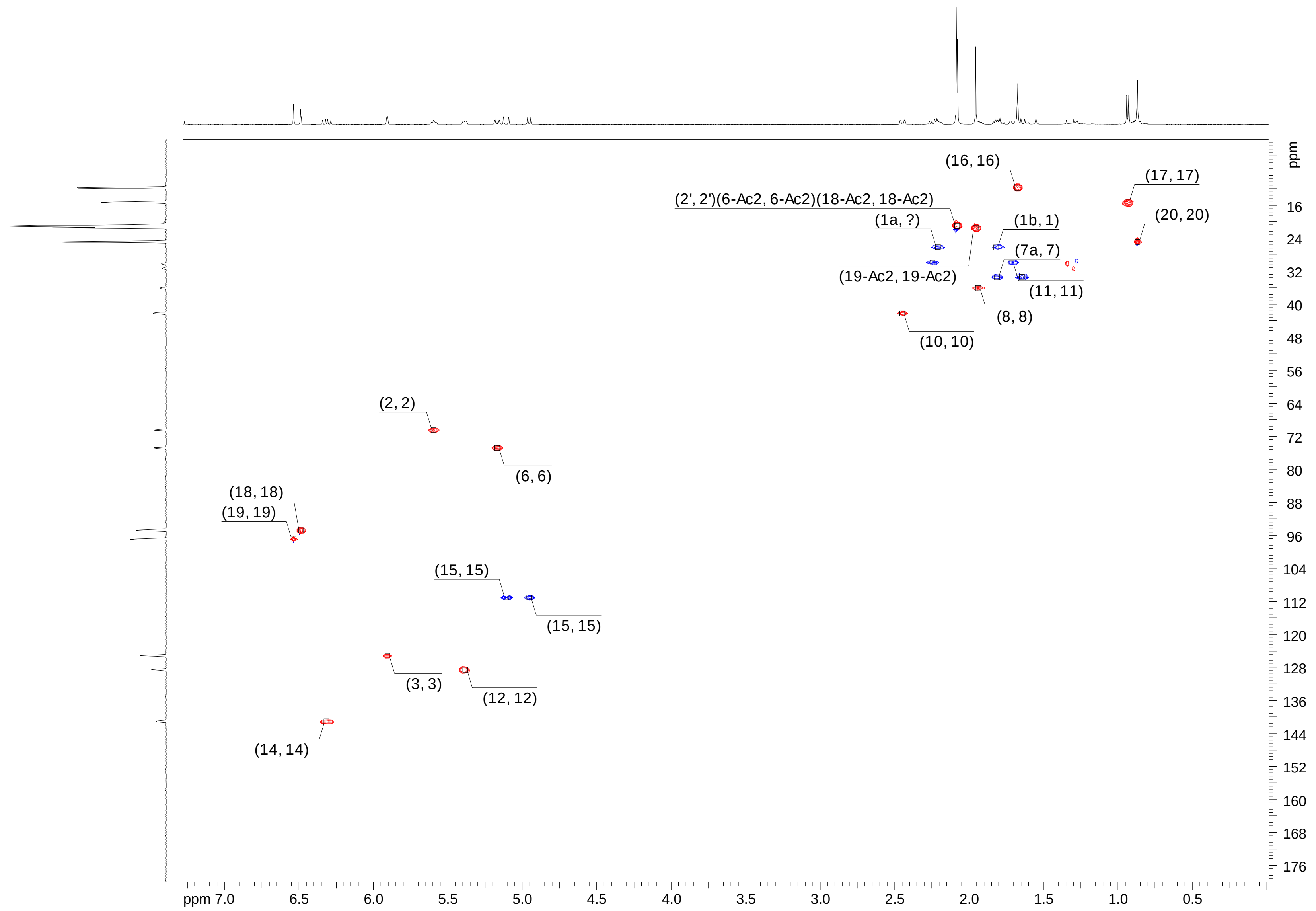

Figure S 19. HSQC spectrum of compound **7** in $CDCl_3$ (500 MHz).

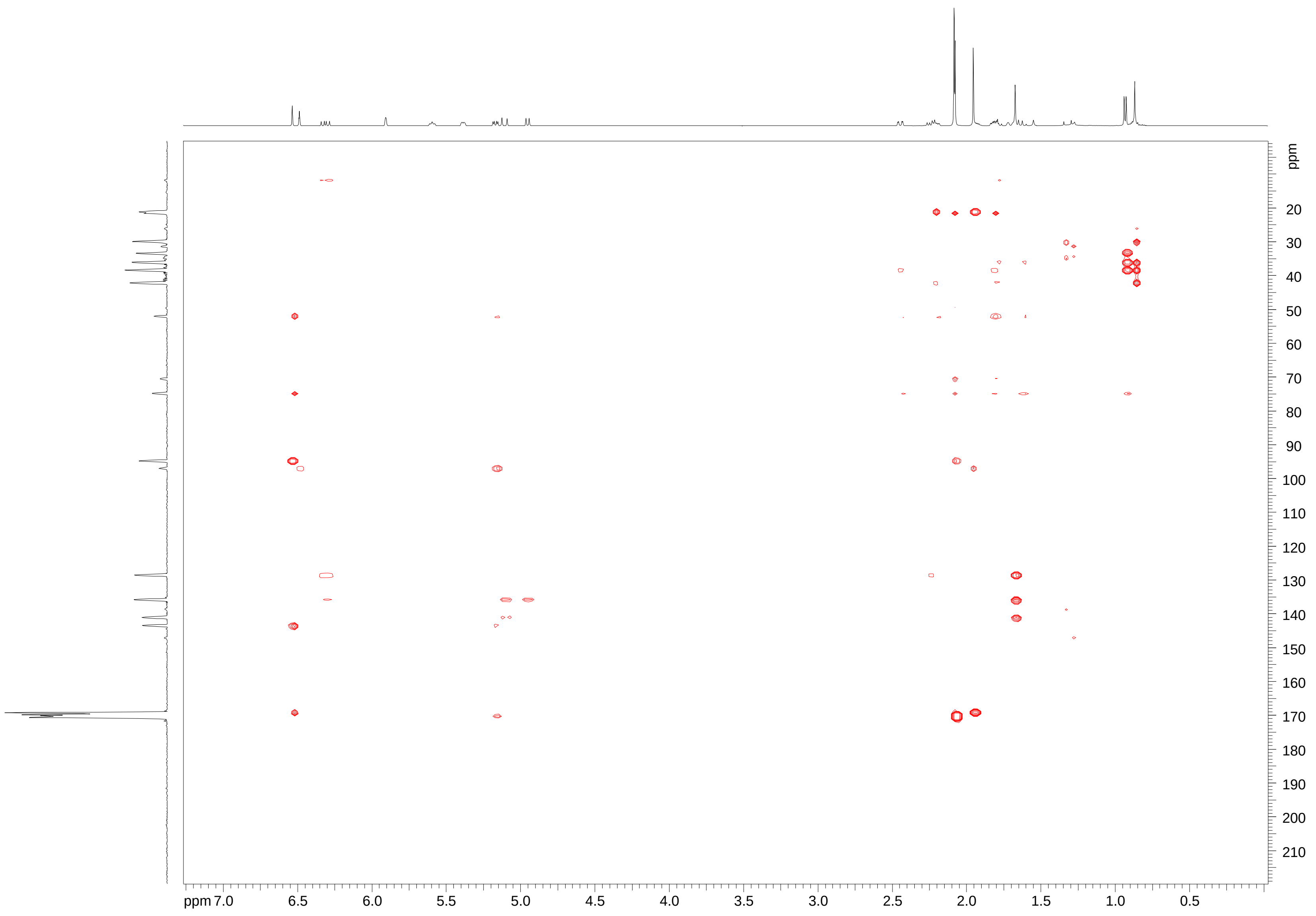

Figure S 16. HMBC spectrum of compound **7** in $CDCl_3$ (500 MHz).

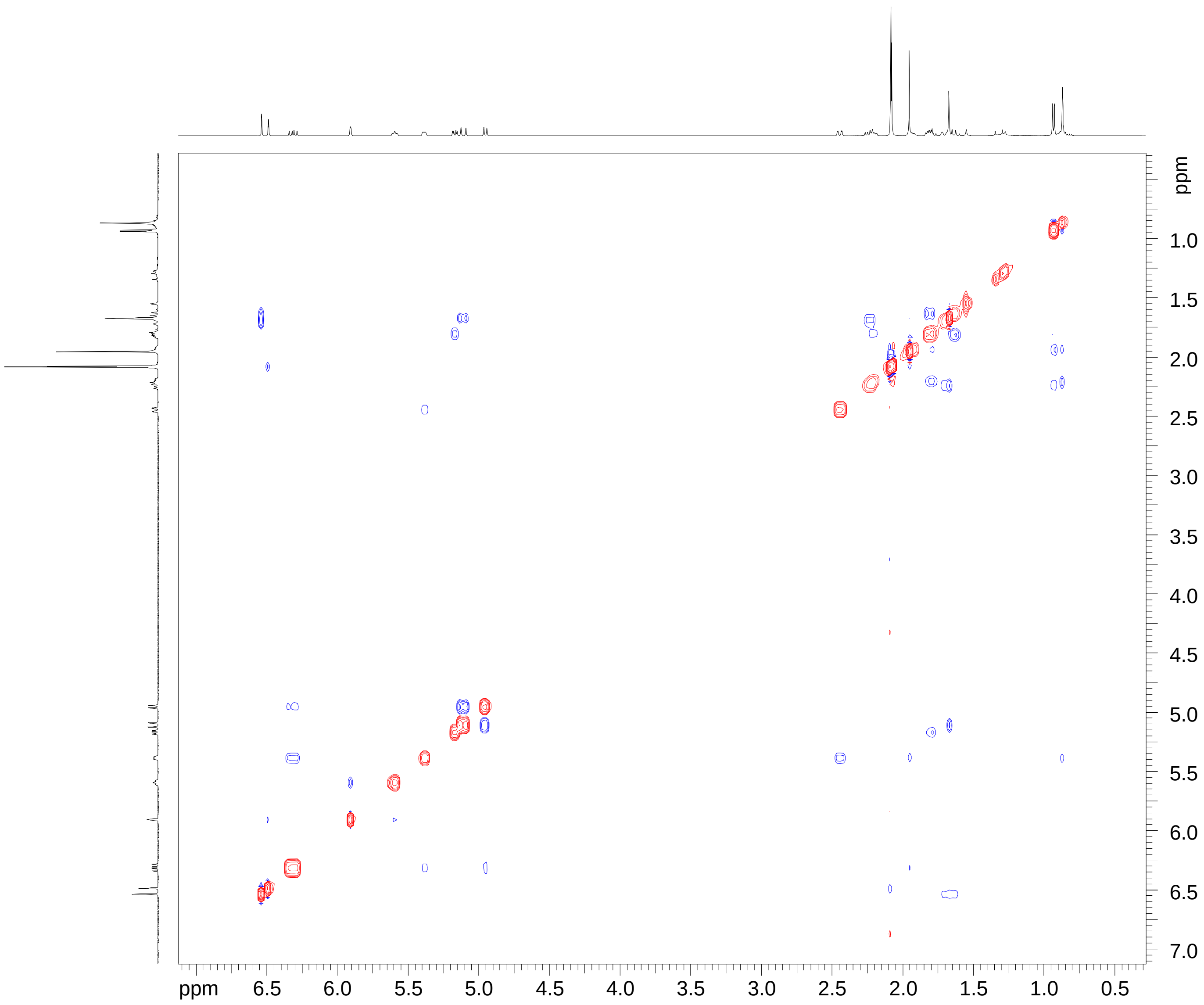

Figure S 17. ^{1}H-^{1}H NOESY spectrum of compound **7** in $CDCl_3$ (500 MHz).

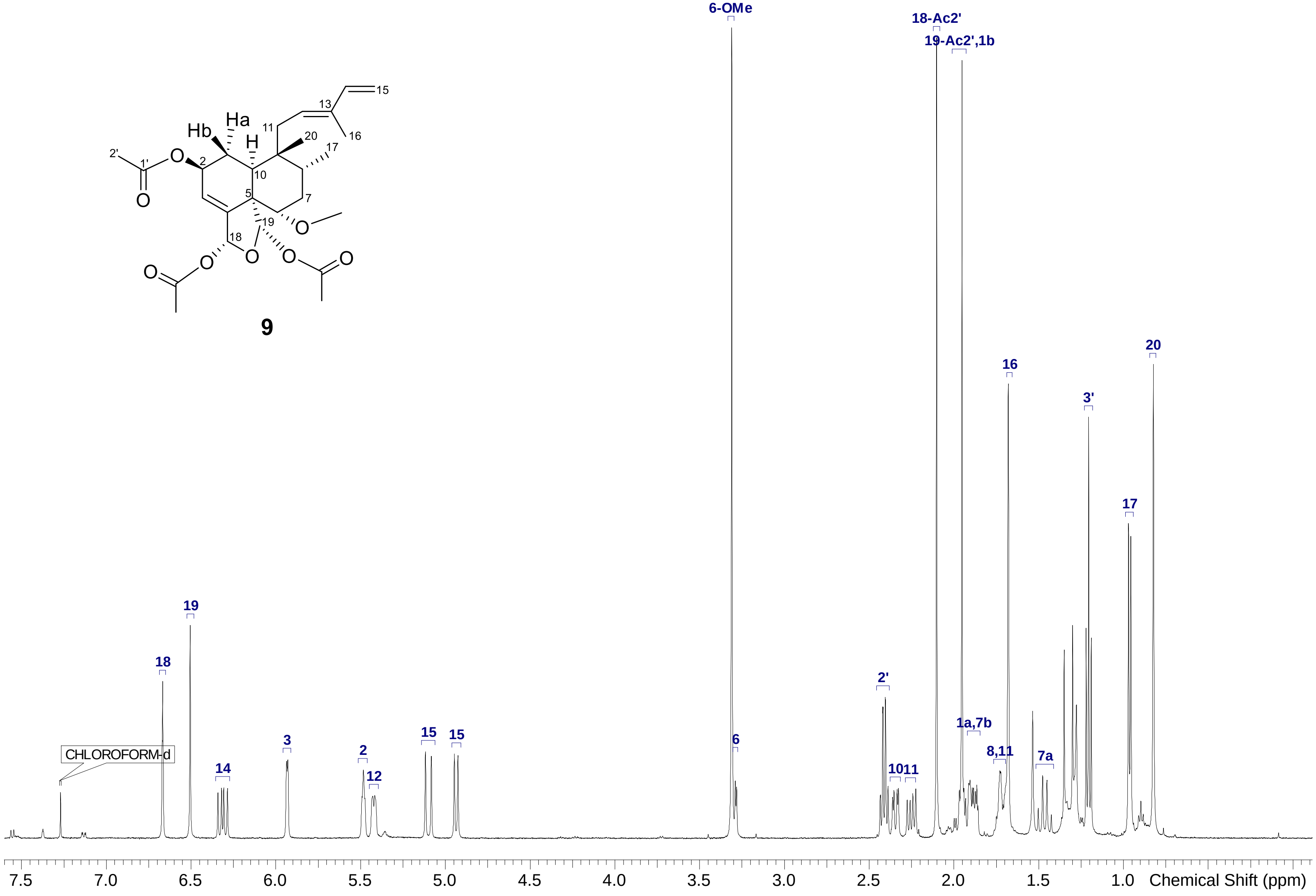

Figure S 22. ^{1}H NMR spectrum of compound **9** in $CDCl_3$ (500 MHz).

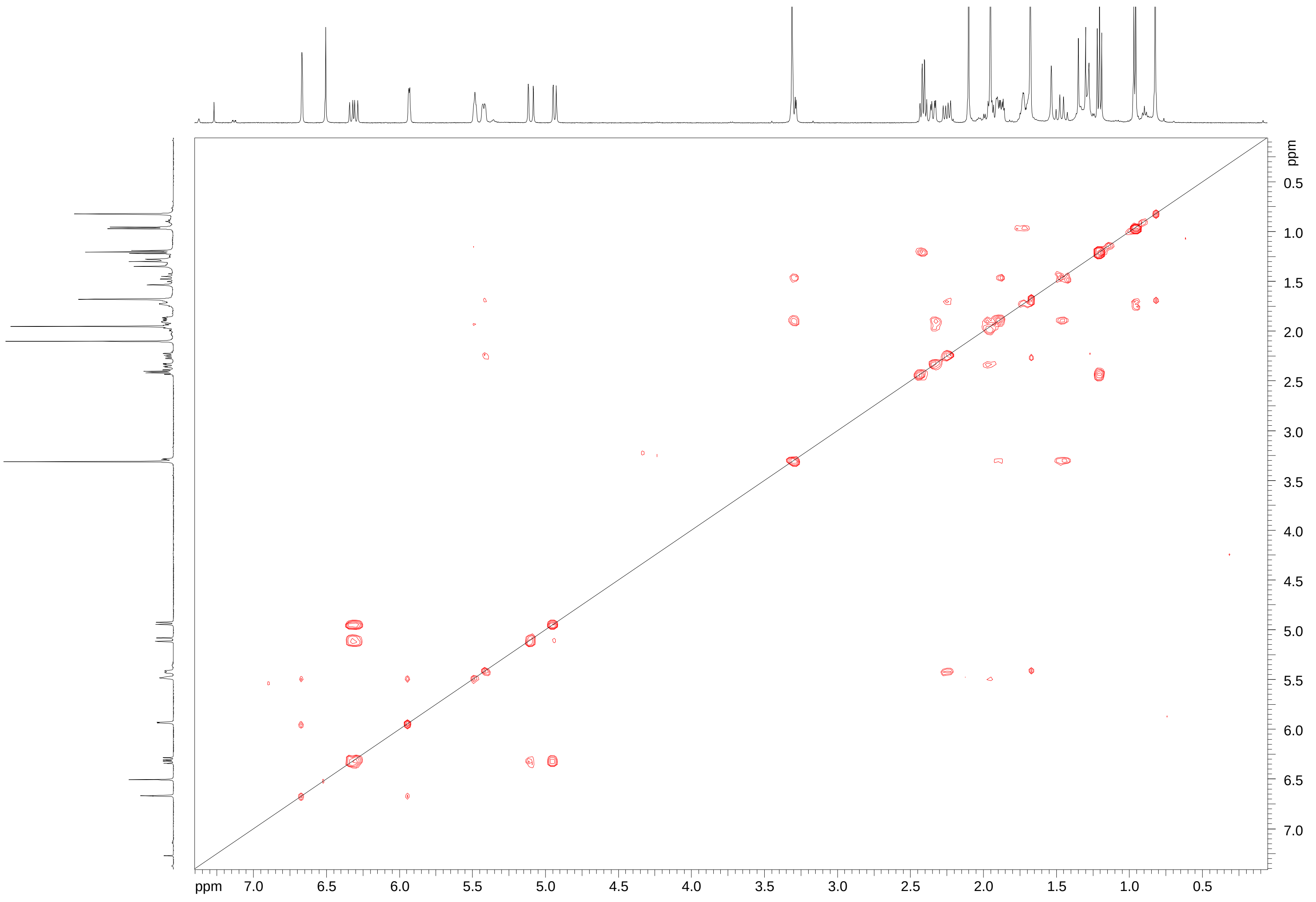

Figure S 18. ^{1}H-^{1}H COSY spectrum of compound **9** in $CDCl_3$ (500 MHz).

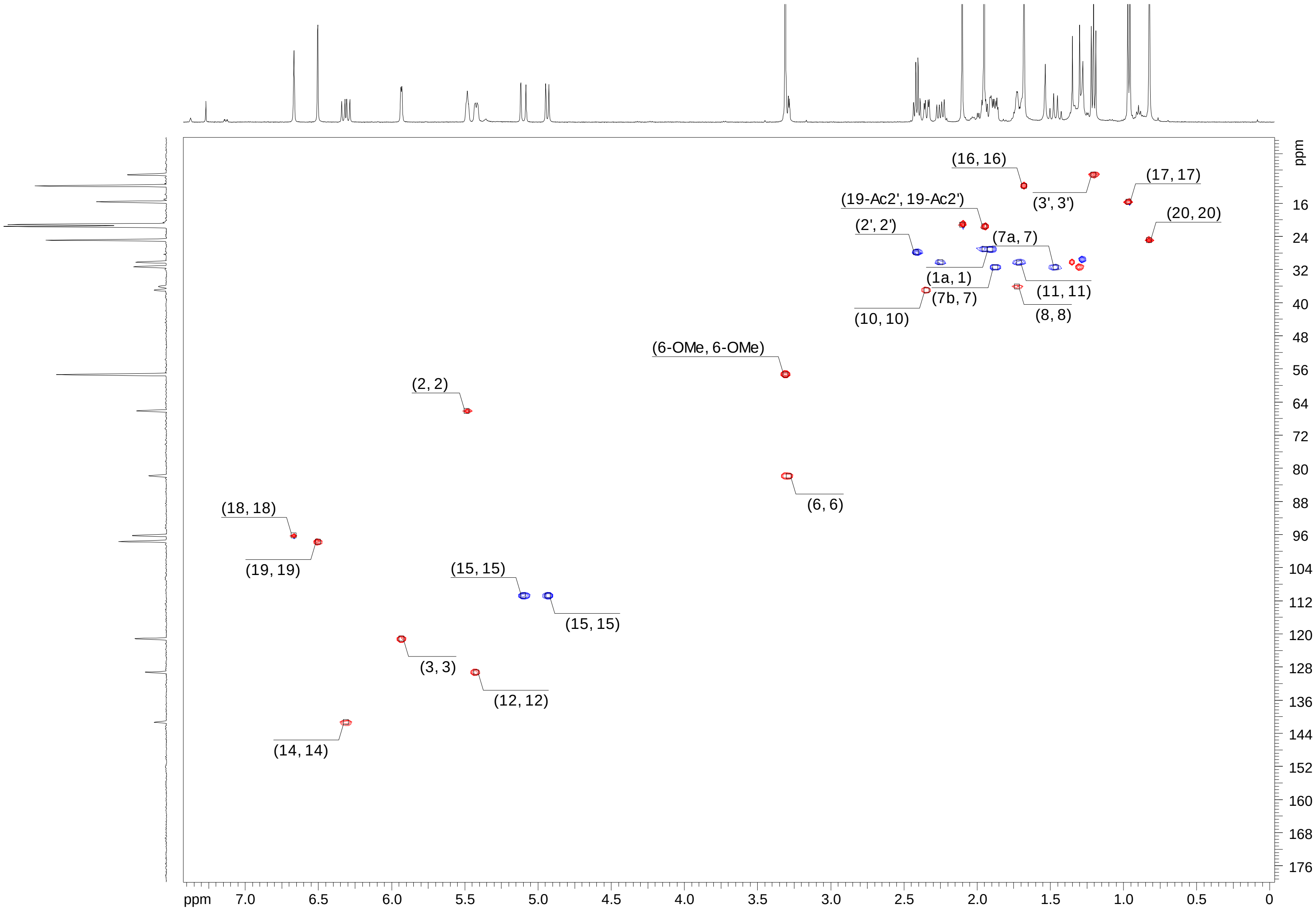

Figure S 19. HSQC spectrum of compound **9** in $CDCl_3$ (500 MHz).

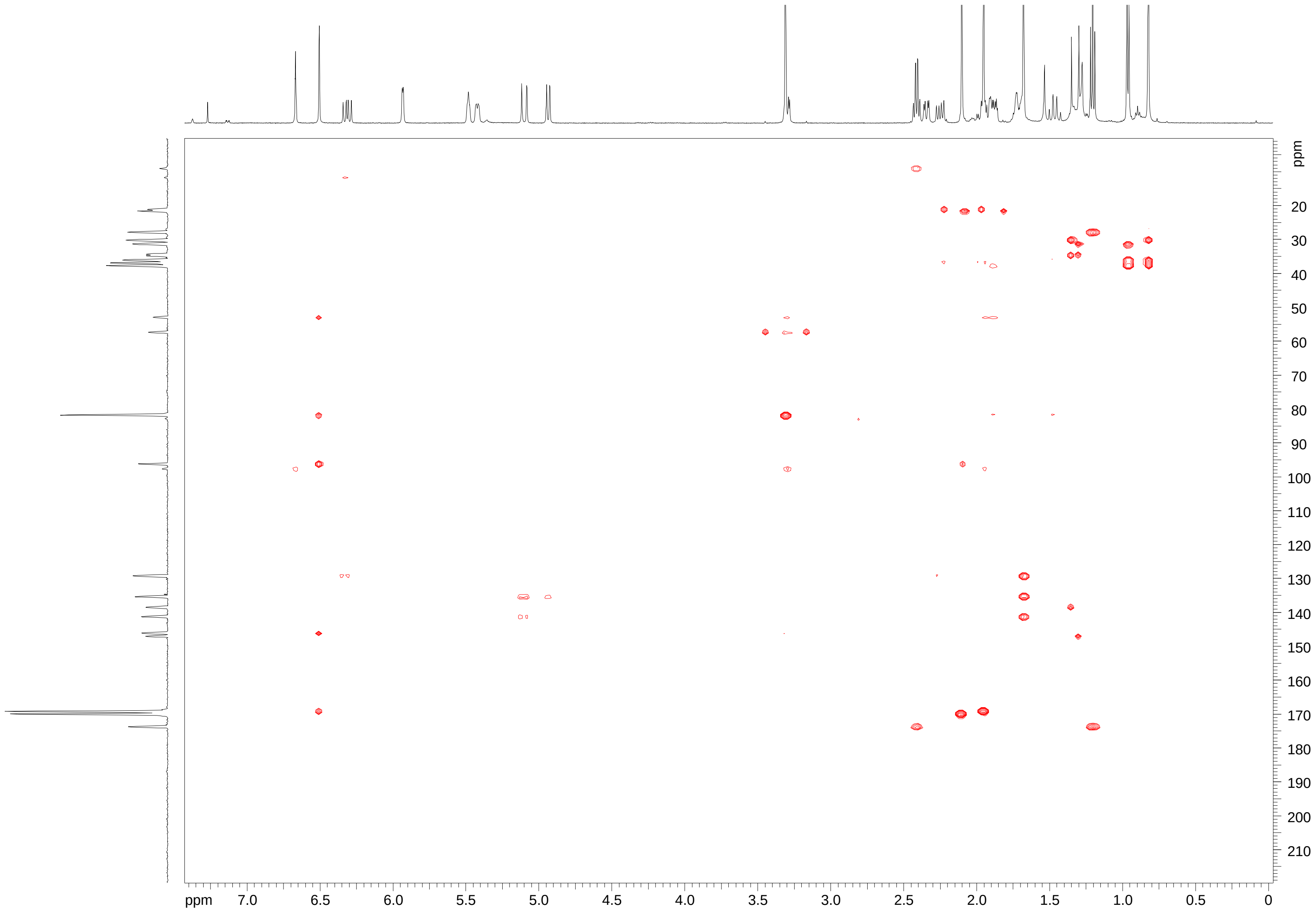

Figure S 2520. HMBC spectrum of compound **9** in CDCl3 (500 MHz).

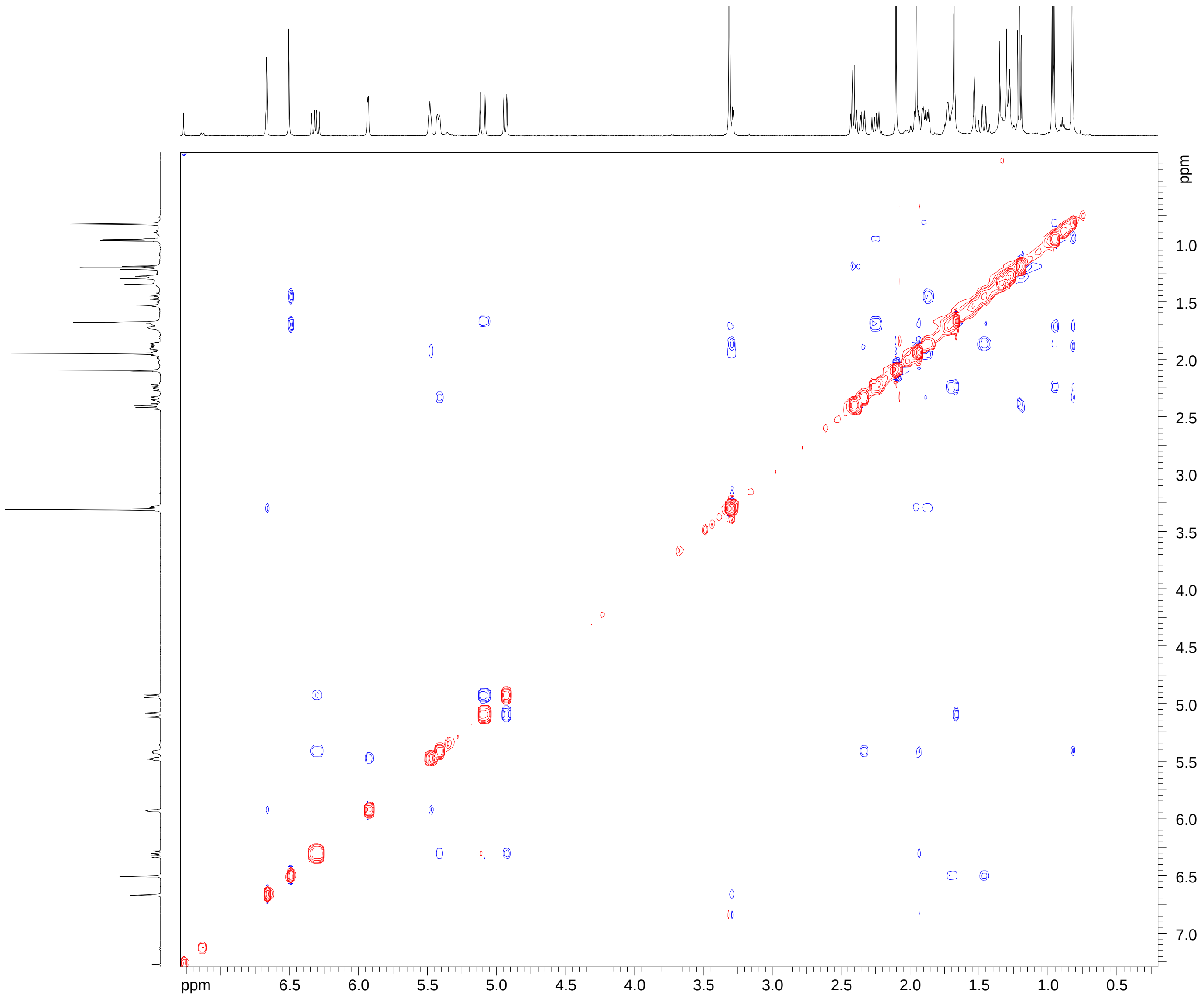

Figure S 21. ^{1}H-^{1}H NOESY spectrum of compound **9** in $CDCl_3$ (500 MHz).

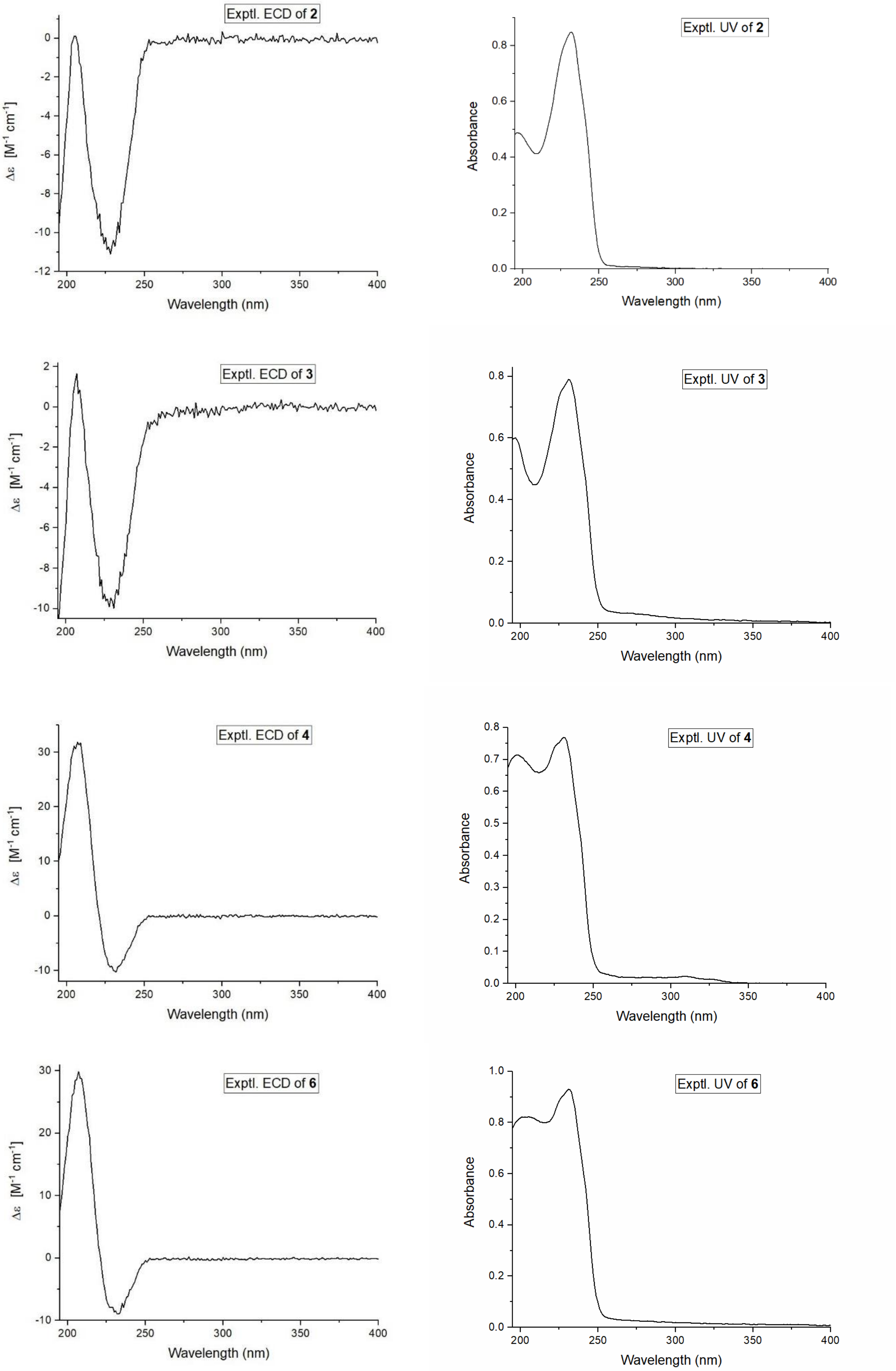

Figure S 227. Experimental ECD and UV spectra of compounds **2-4** and **6** in CH_3CN.

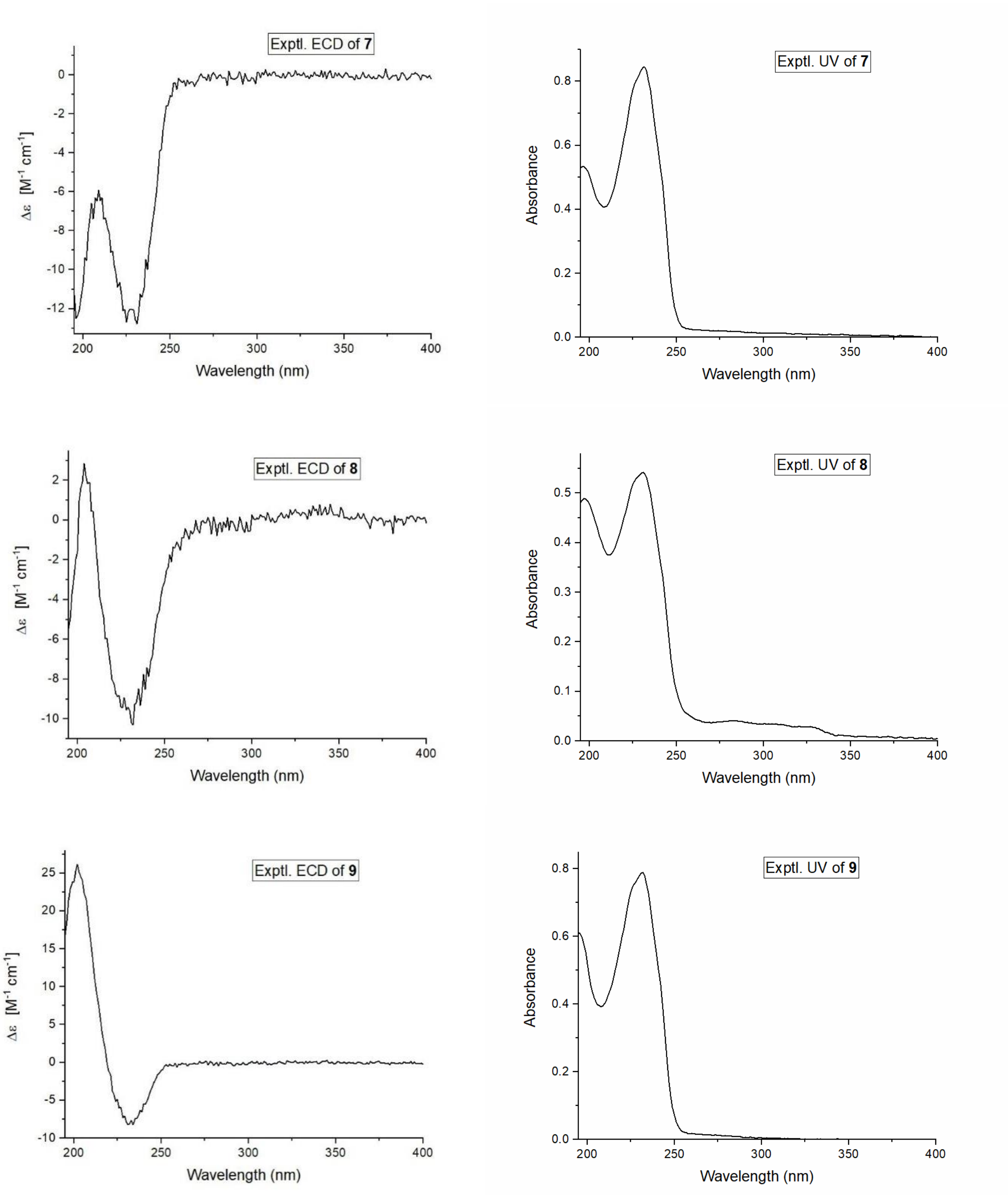

Figure S 23. Experimental ECD and UV spectra of compounds **7 - 9** in CH_3CN.

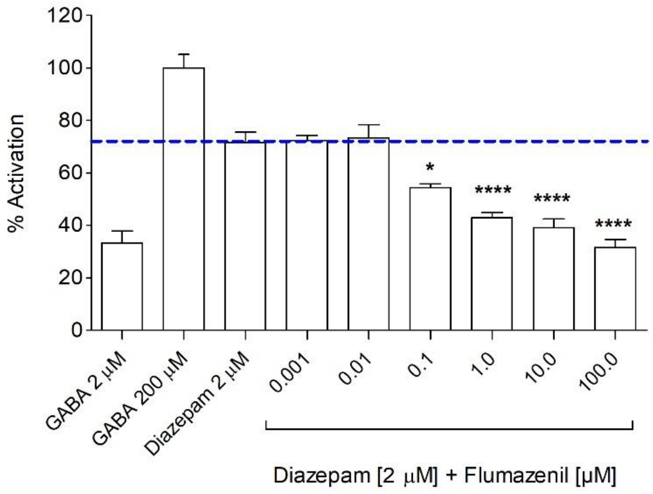

Figure S 24. Percentage of activation for 2 µM diazepam with increasing concentrations of flumazenil (in presence of 2 µM GABA), along with 2 µM GABA (control), 200 µM GABA (100%), and 2 µM diazepam (in presence of 2 µM GABA), (n = 4, mean ± SEM). Final DMSO concentration in the assay was 0.2%. The * and **** above the bars indicate statistical significance with $p \leq 0.05$, and $p \leq 0.0001$, respectively.

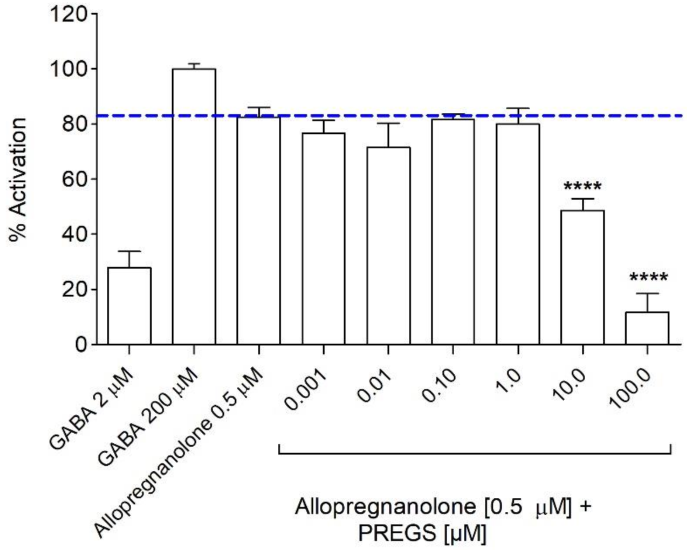

Figure S 25. Percentage of activation for 0.5 µM allopregnanolone with increasing concentrations of PREGS (in presence of 2 µM GABA), along with 2 µM GABA (control), 200 µM GABA (100%), and 0.5 µM allopregnanolone (in presence of 2 µM GABA) (n = 4, mean ± SEM). Final DMSO concentration in the assay was 0.2%. The **** above the bars indicate statistical significance with $p \leq 0.0001$.

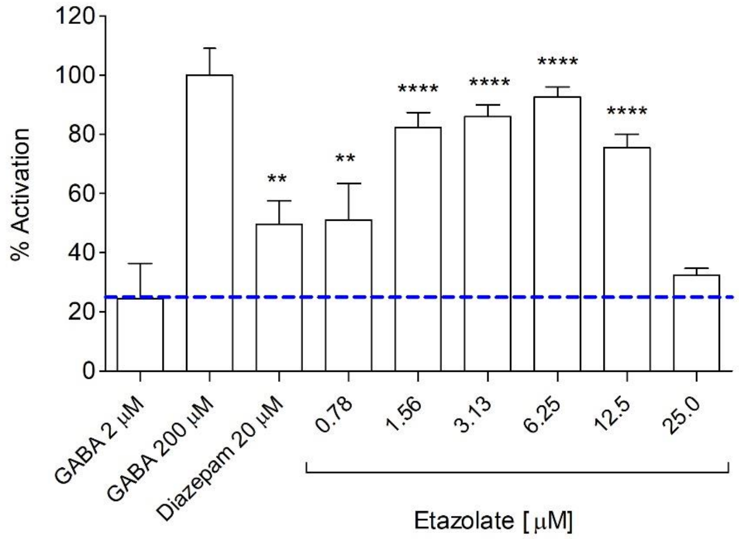

Figure S 26. Percentage of activation for increasing concentrations of etazolate (in presence of 2 µM GABA), along with 2 µM GABA (control), 200 µM GABA (100%), and 20 µM diazepam (in presence of 2 µM GABA), ($n = 4$, mean ± SEM). Final DMSO concentration in the assay was 0.2%. The ** and **** above the bars indicate statistical significance with $p \leq 0.01$, and $p \leq 0.0001$, respectively.

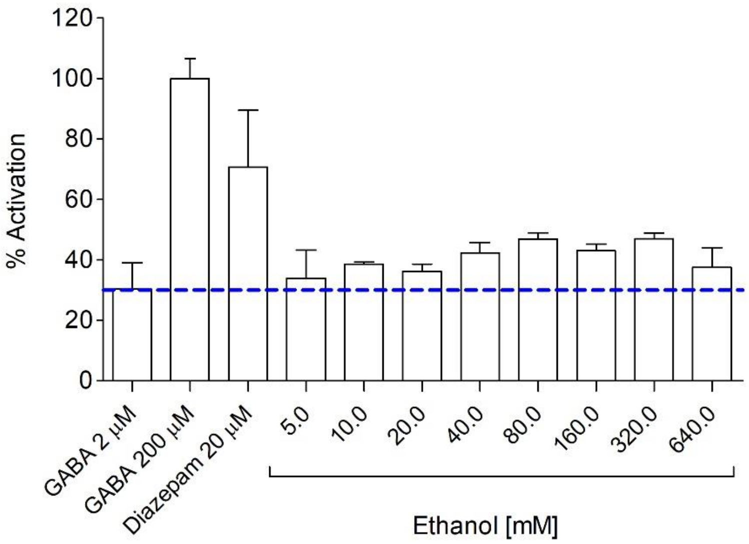

Figure S 27. Percentage of activation for increasing concentrations of ethanol (in presence of 2 µM GABA), along with 2 µM GABA (control), 200 µM GABA (100%), and 20 µM diazepam (in presence of 2 µM GABA), (n = 4, mean ± SEM).

A

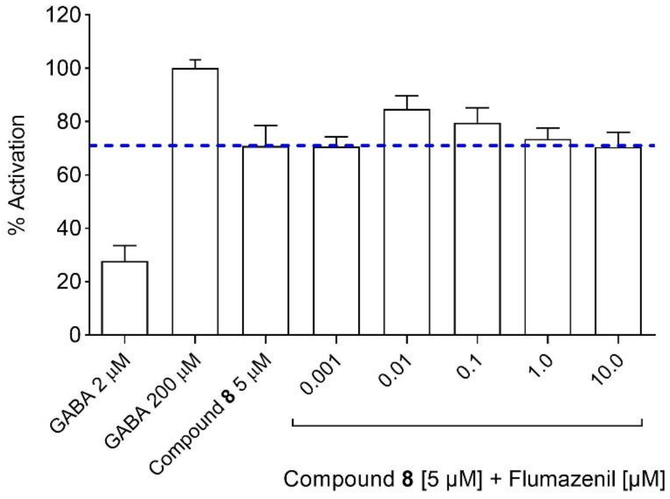

B

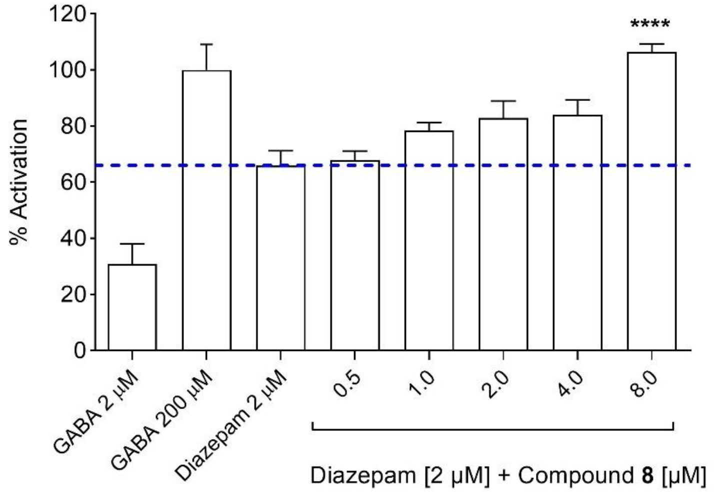

Figure S 33. Percentage of activation by (**A**) **8** (5 μM) and increasing concentrations of flumazenil (in presence of 2 μM GABA), and (**B**) diazepam (2 μM, in the presence of 2 μM GABA) and increasing concentrations of **8** (in presence of 2 μM GABA), together with 2 μM GABA (control), 200 μM GABA (100%), and 2 μM diazepam (in presence of 2 μM GABA; positive control), (n = 4, mean ± SEM). Final DMSO concentration in the assays was 0.2%. The **** above the bars indicate statistical significance with $p \leq 0.0001$.

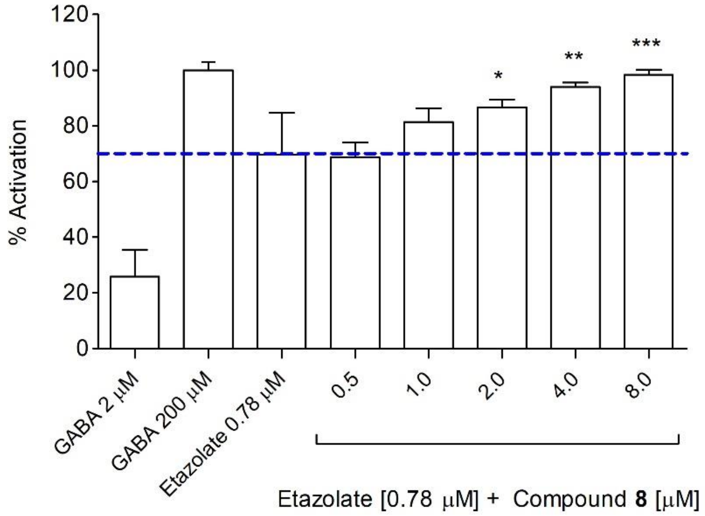

Figure S 34 Percentage of activation by etazolate (0.78 μM) and increasing concentrations of 8 (in presence of 2 μM GABA), along with 2 μM GABA (control), 200 μM GABA (100%), and 0.78 μM etazolate (in presence of 2 μM GABA; positive control), (n = 4, mean ± SEM). Final DMSO concentration in the assay was 0.2%. The *, ** and *** above the bars indicate statistical significance with $p \leq 0.05$, $p \leq 0.01$ and $p \leq 0.001$, respectively.

A

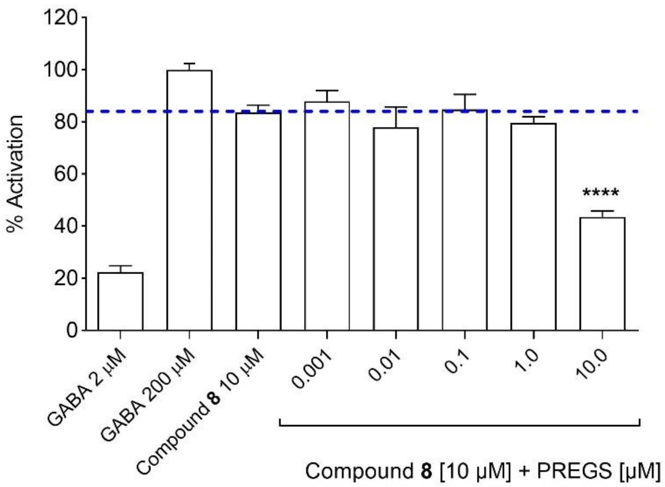

B

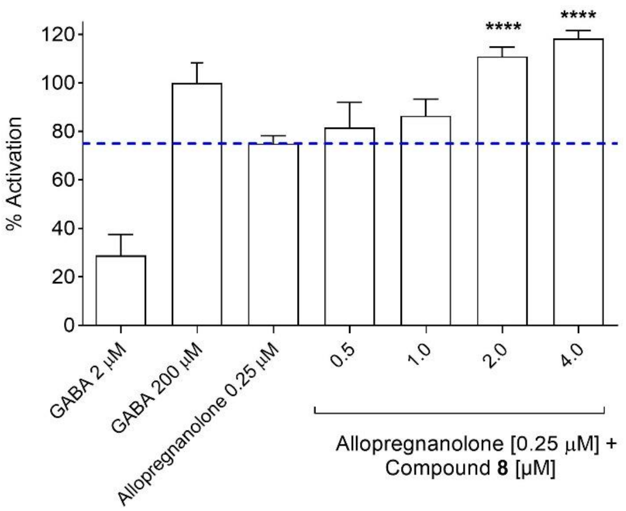

Figure S 35. Percentage of activation (**A**) with compound **8** (10 μM) and increasing concentrations of PREGS (in presence of 2 μM GABA), and (**B**) with allopregnanolone (0.25 μM) and increasing concentrations of **8** (in presence of 2 μM GABA), along with 2 μM GABA (control), 200 μM GABA (100%), and 0.25 μM allopregnanolone (in presence of 2 μM GABA; positive control) (n = 4, mean ± SEM). Final DMSO concentration in the assay was 0.2%. The **** above the bars indicate statistical significance with $p \leq 0.0001$.
